# Reconstructing a hydrogen-driven microbial metabolic network in Opalinus Clay rock

**DOI:** 10.1038/ncomms12770

**Published:** 2016-10-14

**Authors:** Alexandre Bagnoud, Karuna Chourey, Robert L. Hettich, Ino de Bruijn, Anders F. Andersson, Olivier X. Leupin, Bernhard Schwyn, Rizlan Bernier-Latmani

**Affiliations:** 1 Ecole Polytechnique Fédérale de Lausanne, Environmental Microbiology Laboratory, Station 6, Lausanne CH-1015, Switzerland; 2 Oak Ridge National Laboratory, Chemical Sciences Division, Oak Ridge, Tennessee 37831, USA; 3 Bioinformatics Infrastructure for Life Sciences (BILS), Stockholm 171 65, Sweden; 4 KTH Royal Institute of Technology, Science for Life Laboratory, School of Biotechnology, Division of Gene Technology, Stockholm 171 65, Sweden; 5 Nagra, Wettingen CH-5430, Switzerland

## Abstract

The Opalinus Clay formation will host geological nuclear waste repositories in Switzerland. It is expected that gas pressure will build-up due to hydrogen production from steel corrosion, jeopardizing the integrity of the engineered barriers. In an *in situ* experiment located in the Mont Terri Underground Rock Laboratory, we demonstrate that hydrogen is consumed by microorganisms, fuelling a microbial community. Metagenomic binning and metaproteomic analysis of this deep subsurface community reveals a carbon cycle driven by autotrophic hydrogen oxidizers belonging to novel genera. Necromass is then processed by fermenters, followed by complete oxidation to carbon dioxide by heterotrophic sulfate-reducing bacteria, which closes the cycle. This microbial metabolic web can be integrated in the design of geological repositories to reduce pressure build-up. This study shows that Opalinus Clay harbours the potential for chemolithoautotrophic-based system, and provides a model of microbial carbon cycle in deep subsurface environments where hydrogen and sulfate are present.

## Abstract (web summary)

Hydrogen build-up in geological nuclear waste repositories poses risks, but it may be alleviated by H_2_ consumption by deep subsurface microbial communities. Here, the authors inject H_2_ in a borehole and use metagenomics and metaproteomics to identify a carbon cycle driven by autotrophic H_2_ oxidizers.

## Graphical abstract

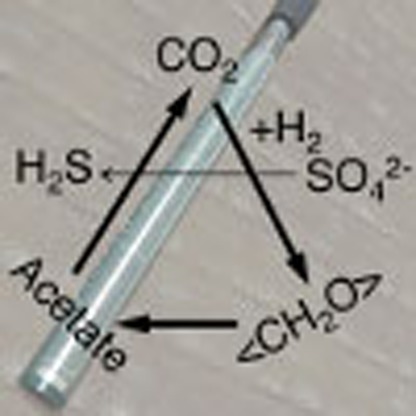

A significant fraction of living microorganisms is found in the deep terrestrial subsurface^1^,^2^, in zones hydrologically disconnected from the Earth's surface^3^. Such microbial communities may not directly rely on sunlight as an energy source, but rather on geogenic reduced compounds, such as hydrogen gas (H_2_), when buried organic matter is absent^4^,^5^. Hydrogen is produced by the serpentinization (anoxic oxidation) of mafic and ultramafic rocks^6^ and the radiolysis of water^7^. Hydrogen is a key metabolic compound in many anoxic ecosystems and its oxidation has been suggested to support deep subsurface lithoautotrophic microbial ecosystems^8^,^9^,^10^,^11^,^12^,^13^.

Because of the difficulty of access, there is limited understanding of microbial systems in the deep subsurface^14^. In these environments, functional diversity of microorganisms has been assessed by several sequencing methods, such as 16S rRNA gene sequencing^10^,^15^, unassembled metagenomic reads^12^, assembled metagenomic contigs^16^,^17^ and metagenomic-assembled genomes (MAGs) from low-11,18 and high-diversity microbial communities^13^. MAGs represent a significant contribution, because they allow for the characterization of the putative metabolism of individual organisms, and the reconstruction of inferred metabolic networks at the community level. But all of the above studies were based on the presence of genetic material, which only represents the potential for a given metabolism. In order to pinpoint extant metabolic processes, metatranscriptomic and/or metaproteomic analyses are needed, because they can reveal the presence of active functions (by detecting messenger RNAs and proteins, respectively).

Microbiological studies of the deep subsurface are relevant for various reasons. In particular, microbial processes influence the geochemistry of groundwater and oil reservoirs, impacting the feasibility of resource extraction. An additional motivation is related to nuclear waste management. Several European countries have opted to dispose of nuclear waste in deep geological repositories, where the waste will be isolated for periods up to one million years, until a reasonable radioactivity level is reached^19^. The design of such systems requires a detailed understanding of the major processes (physical, chemical and biological) that could take place. For instance, anoxic corrosion of steel will release H_2_ that could increase the pressure in a closed repository^20^, jeopardizing the integrity of engineered barriers. On the other hand, H_2_ is an energy and electron source for microorganisms. Thus, microbial activity in a repository is expected to have a beneficial impact, by reducing H_2_ overpressure in this confined environment^21^.

In order to test this hypothesis, we use the Opalinus Clay rock formation, which is the current candidate for hosting a deep geological repository in Switzerland^22^. This rock is characterized by a small pore size distribution (10–20 nm on average), the presence of reduced mineral species (siderite, pyrite)^23^, sulfate (SO_4_^2−^) as a porewater solute (10–25 mM)^24^, solid phase organic matter (1.5% w/w)^24^ and dissolved organic matter (200 μM acetate)^25^. Injection of H_2_ in a borehole in Opalinus Clay, 300 m below the surface, combined with monitoring of borehole water captured the resulting chemical and biological changes as well as the microbial metabolic networks under repository-relevant conditions.

Our study demonstrates that Opalinus Clay harbours a microbial community that is able to efficiently oxidize H_2_, which is beneficial for the safety of deep geological nuclear waste repositories, through the reduction of pressure build-up caused by anoxic steel corrosion. While the H_2_ concentrations in this study are unrealistic for natural deep subsurface environments (because they are too high), they are relevant for repositories, and promote microbial biomass build-up, allowing a metaproteomic analysis. Used jointly with metagenomic sequencing and genome binning, this technique highlights active metabolic pathways. The metabolic network described here, a carbon cycle based on chemolithoautotropy, is of general interest for deep subsurface microbiology, and can serve as a model for understanding autotrophic microbial communities relying on H_2_, carbon dioxide (CO_2_) and sulfate.

## Results

### Chemical and biological changes

**Figure 1:**
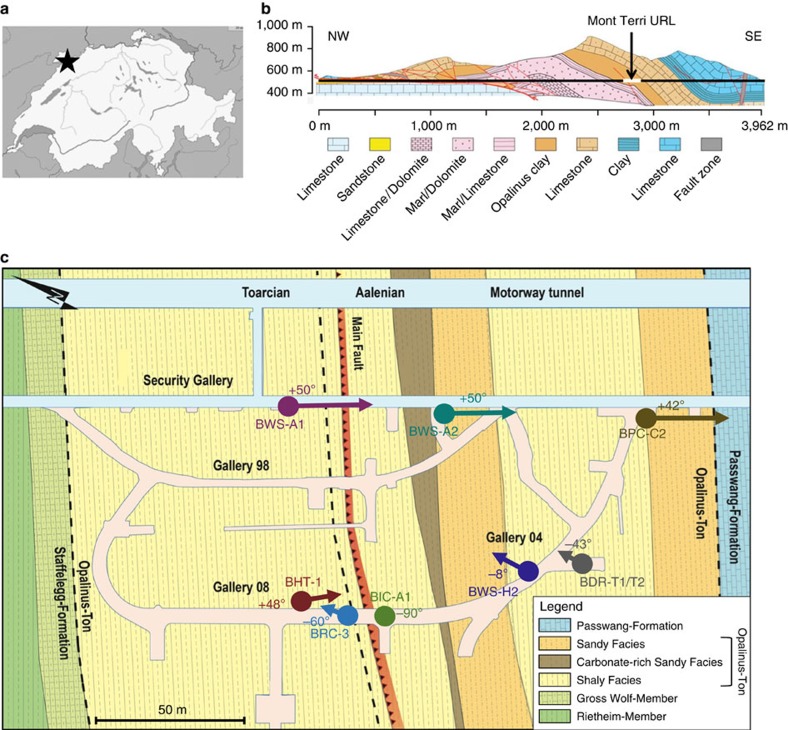
Situation of the Mont Terri Underground Rock Laboratory (URL) and borehole emplacements. (**a**) Location of the Mont Terri URL in Switzerland. (**b**) Cross-section of the motorway tunnel of Mont Terri, whose security gallery was used for excavating the URL. (**c**) Location of the boreholes sampled in this study. The circles indicate the position of the boreholes opening and the angles indicate the boreholes orientation compared to the ground (a negative angle means a descending borehole). The borehole used in the experiment is BRC-3. Modified from Swisstopo^26^.

**Figure 2:**
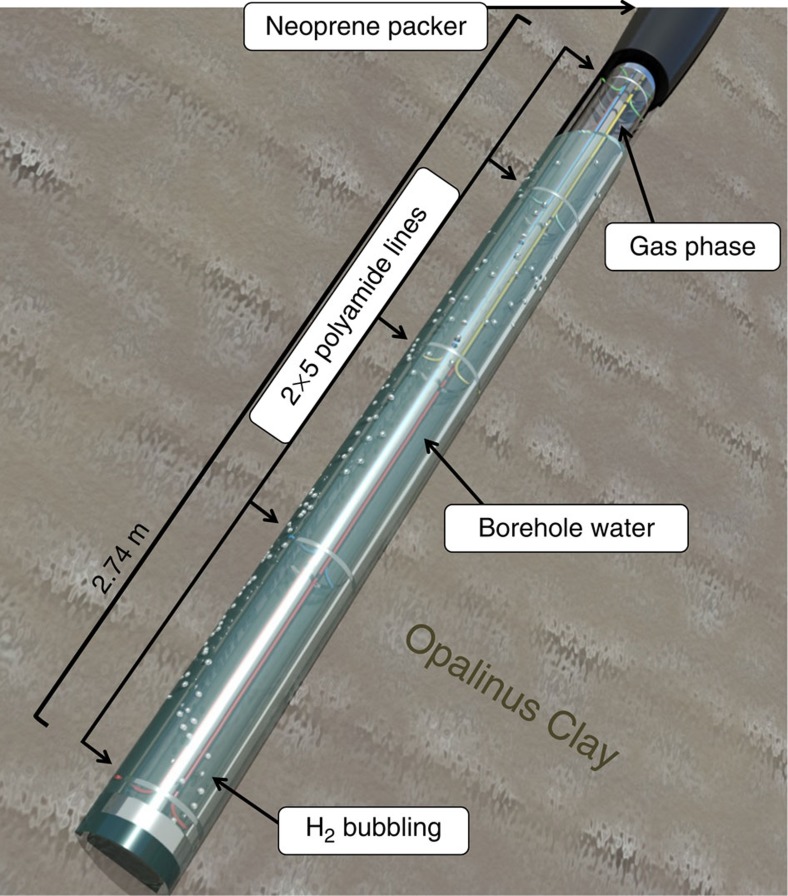
An artist's rendition of borehole equipment. For better clarity, the polyvinyl chloride screen is not shown. Neoprene packer isolates the borehole water from the oxic atmosphere of the Underground Rock Laboratory galleries. Polyamide lines allow to sample borehole water, and to inject APW and H_2_ (which is creating a headspace) at different depths.

**Figure 3:**
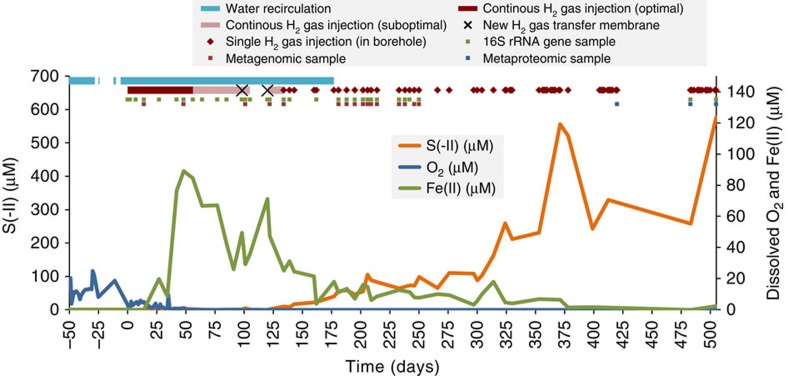
Timeline of the experiment and the chemical evolution of its borehole water. Concentrations of dissolved O_2_ are indicated with the blue line (right y-axis), Fe(II) with the green line (right y-axis) and S(-II) with the orange line (left y-axis) in borehole water, starting 50 days before the first H_2_ injection that occurs at day 0. Initially, H_2_ injection was done through a small reservoir at the surface via a gas permeable membrane when recirculating porewater. After 50 days, H_2_ input was suboptimal because of membrane clogging (Supplementary Fig. 2). It is the reason why, starting day 134, H_2_ was regularly injected directly in the borehole, thus creating a gas phase. Shortly thereafter, it was also decided to stop porewater circulation because of H_2_ loss at the peristaltic pump. The experiment procedure is indicated at the top of the plot: the light blue line shows when water was recirculated, the dark red line shows when H_2_ was optimally delivered from the surface, the light red line shows when H_2_ was sub-optimally delivered from surface, the dark crosses show when the H_2_-permeable membrane was changed, and red diamonds show when H_2_ was injected directly into the borehole. Samples are also indicated at the top of the plot: green squares show samples for 16S rRNA gene sequencing, red squares show samples for metagenomic sequencing, and blue square show samples for metaproteomic analysis.

In order to mimic repository-relevant conditions, we performed an *in situ* experiment 300 m below the surface, in the Mont-Terri Underground Rock Laboratory^26^ (St-Ursanne; Switzerland; Fig. 1). We injected H_2_ in a borehole in Opalinus Clay (Fig. 2), and monitored the chemical and biological changes for over 500 days. Geochemical monitoring of borehole water revealed that oxygen (O_2_) (resulting from drilling) was consumed rapidly (within 1 month), leading to anoxic conditions (Fig. 3) after the onset of H_2_ amendment. This occurrence had limited consequences on the long-term experiment. This is because ferric iron (Fe(III)), produced by exposure to O_2_, was reduced back to ferrous iron (Fe(II)) early in the experiment once the conditions returned to anoxia (Fig. 3). Additionally, the indigenous community of obligate anaerobes, if present, was steadily delivered into the borehole through the influx of porewater (∼20 ml per day). Within 150 days of the first H_2_ amendment, soluble sulfide (mainly HS^−^) accumulated to concentrations up to 600 μM, highlighting the dominance of sulfate reduction (Fig. 3). Sulfate is naturally present in the porewater at *ca*. 24 mM and slowly decreased over time, due to microbial sulfate reduction and dilution by artificial porewater (APW; Supplementary Fig. 1). Indeed, the concentration of sulfate in APW that was injected until day 317 was lower than that in natural borehole water (Supplementary Table 1). H_2_ was consumed concomitantly with SO_4_^2−^ and served as the electron donor in its reduction (Supplementary Fig. 2).

**Figure 4:**
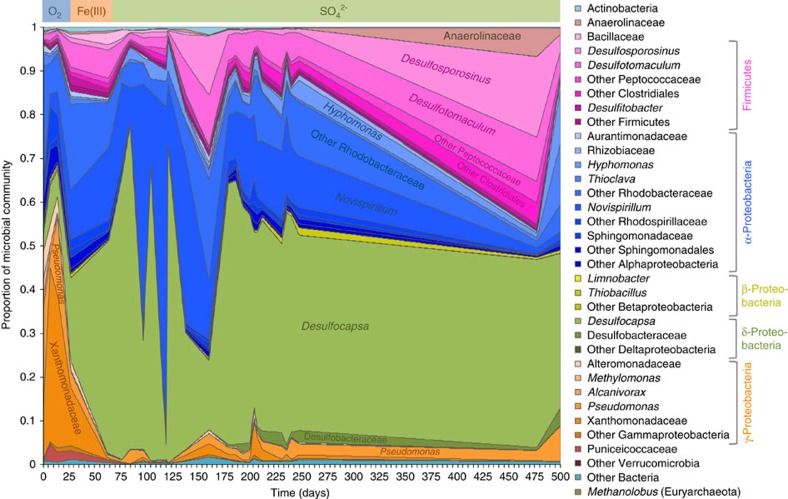
Temporal variation of microbial community composition of the borehole water. This community was assessed with amplicon sequencing of the V4 region of the 16S rRNA gene. The first sample at day 0 was recovered prior to the first H_2_ injection. The coloured bar at the top of the plot indicates the final electron acceptor in the different redox regimes (Fig. 3).

An increase in planktonic cell density was coincident with the reduction of O_2_, Fe(III), then SO_4_^2−^, coupled with H_2_ oxidation (Supplementary Fig. 3), suggesting that these processes are biological. This increase in cell numbers was concomitant with a decrease in dissolved CO_2_ concentration in the porewater (Supplementary Fig. 4), also suggesting autotrophic growth. Sequencing of the 16S rRNA gene V4 region of DNA extracted from borehole water containing suspended clay particles revealed a microbial community dominated by bacteria, whose composition fluctuated initially but remained stable once a sulfate-reducing regime was established (Fig. 4; Supplementary Data 1). Aerobic Gammaproteobacteria^27^ initially represented about 60% of the microbial community, but were largely replaced by metabolically versatile Alphaproteobacteria^28^, and Gram-positive and Gram-negative sulfate-reducing bacteria (SRB)^29^,^30^ once O_2_ was depleted.

### Metagenome-assembled genomes recovery

**Figure 5:**
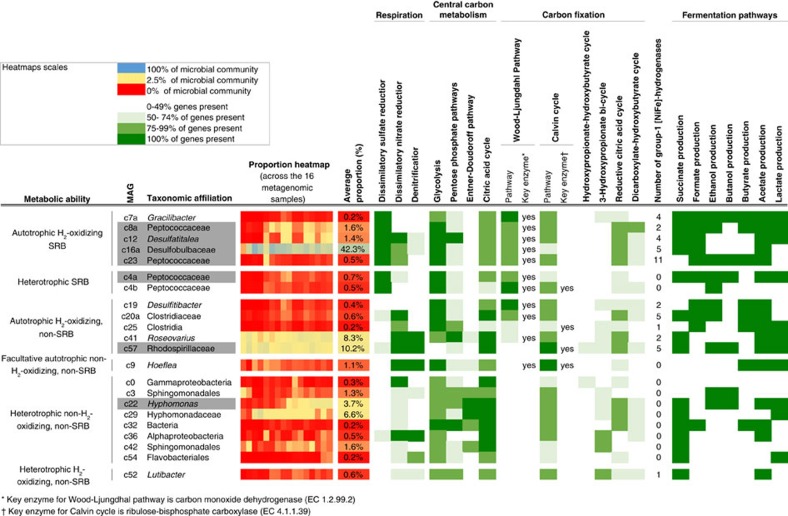
Metabolic abilities of the 22 high-quality MAGs. The completeness of selected metabolic pathways, such as respiratory processes, central carbon metabolism, H_2_ oxidation linked to respiratory processes, carbon fixation and fermentation pathways, is indicated for each genome (green scale). This figure also includes, for each genome, its average proportion and its proportion within each sample (blue, yellow and red scale). The seven MAGs with protein expression information are highlighted in grey shading.

Metagenomic binning allowed the identification of 65 putative genomes (Supplementary Data 2), which is in line with other metagenomic studies of the deep subsurface^13^. Twenty-two bins were found to be free of misassembled or wrongly binned sequence and complete (or nearly complete; Supplementary Table 2)^31^, and represented more than 83% of the microbial community (Fig. 5). Henceforth, these bins will be referred to as MAGs^32^. These MAGs represent a subset of the operational taxonomic units (OTUs), which were defined by 16S rRNA gene sequencing. Indeed, most of the MAGs harbouring a 16S rRNA gene could be linked up to OTUs (Supplementary Table 3). The 16S rRNA gene was missing for some MAGs (c0, c3, c23, c25, c36 and c42), which made their taxonomic affiliation less precise (Supplementary Table 4). Another seven MAGs (c4a, c4b, c8a, c16a, c20a, c32 and c57) represented new genera, as shown by their taxonomic annotation (Supplementary Table 4) and their average nucleotide identity (ANI) with reference genomes (Supplementary Table 5). Overall, the two methods—metagenomic binning and 16S rRNA gene sequencing—described microbial communities of similar composition (Supplementary Fig. 5, Fig. 4).

Metabolic pathways of the 22 abovementioned MAGs were inferred from their protein annotation and resulted in their classification into six categories: (i) autotrophic SRBs able to use H_2_ as an energy and electron source to reduce CO_2_; (ii) heterotrophic SRBs able to oxidize acetate to CO_2_; (iii) autotrophic bacteria unable to reduce sulfate, but able to oxidize H_2_; (iv) facultative autotrophic bacteria unable to reduce sulfate or to oxidize H_2_, expected to exhibit heterotrophic growth *in situ*, (v) heterotrophic bacteria unable to reduce sulfate or to oxidize H_2_, and (vi) heterotrophic bacteria unable to reduce sulfate but able to oxidize H_2_ (Fig. 5).

### Metaproteomic analysis and community-level metabolic network

**Figure 6:**
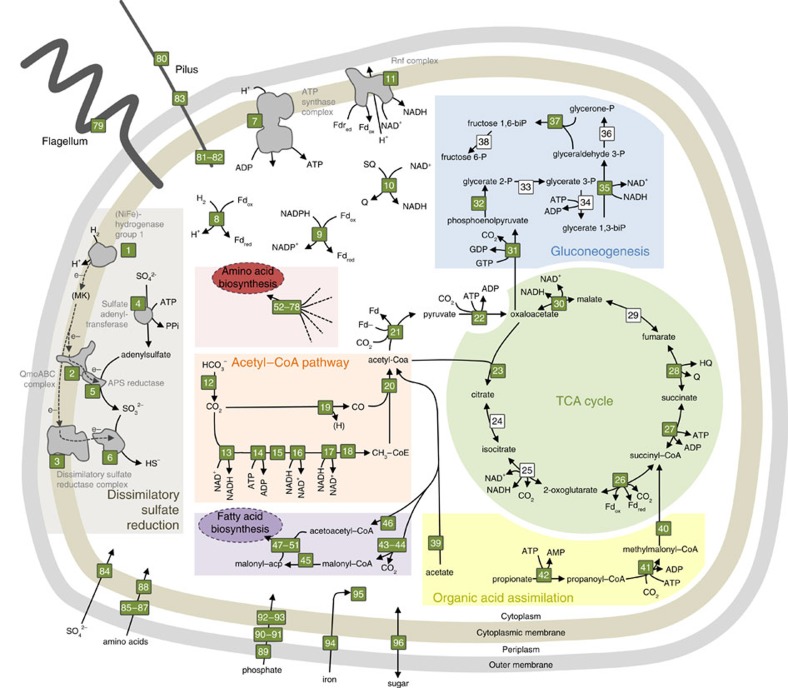
Metabolic pathways of Desulfobulbaceae c16a. Proteomic evidence are indicated with green boxes, and the genomic evidence with white boxes. This microorganism reduces SO_4_^2−^ to HS^−^ using electrons from H_2_ (grey compartment), thus producing a proton gradient across the cytoplasmic membrane. The intracellular proton translocation is coupled to ATP generation. ATP and electrons (the latter being transferred via reduced ferredoxin and NADH/NADPH) can be used to reduce CO_2_ to acetyl-CoA via the reductive acetyl-CoA pathway (orange compartment). Biomass biosynthesis takes place through the tricarboxylic acid cycle (green compartment), and gluconeogenesis (blue compartment). Fatty acids and amino acids biosynthesis is not described in detail, but the proteins involved are listed in purple and red boxes, respectively. Finally, this bacterium can also use acetate and propionate as a source of carbon (yellow compartment). All proteins of this figure are listed in Supplementary Table 7.

**Figure 7:**
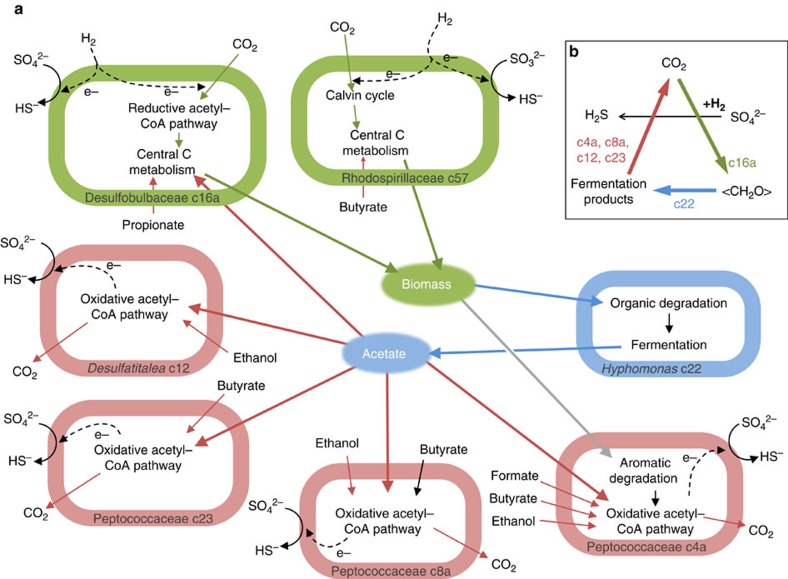
Carbon cycle inferred from metaproteomic data. Carbon fixation is colour-coded in green, fermentation in blue and complete oxidation of fermentation products in red. (**a**) Metabolic interactions between seven microorganisms. Carbon fixation is carried out by a sulfate-reducing bacterium (Desulfobulbaceae c16a) and by a non-sulfate reducing bacterium (Rhodospirillaceae c57). Both use H_2_ as an electron source. All other organisms are heterotrophic. A fermenting bacterium (*Hyphomonas* c22) can oxidize organic macromolecules to acetate, while sulfate-reducing bacteria (Peptococcaceae c4a, c8a, c23 and *Desulfatitalea* c12) oxidizes acetate to CO_2_. All protein data used to build this metabolic interaction are listed in Supplementary Data 6. (**b**) A simplified model of this carbon loop based on the presence of CO_2_, H_2_ and sulfate is illustrated.

Metaproteomic analysis of a sample recovered after 483 days, when the system reached stable sulfate-reducing conditions, uncovered sufficient protein information to decipher metabolic pathways for seven MAGs (c4a, c8a, c12, c16a, c22, c23, c57; Supplementary Tables 6, Supplementary Data 3, 4 and 5), representing more than 60% of the microbial community, and enabling the identification of their metabolic activity. Only MAG c16a contained enough protein information to reconstruct a detailed metabolic map, as presented in Fig. 6. The protein information of the others MAGs was only sufficient to broadly identify their metabolism, as is shown in Fig. 7a. The 16S rRNA analysis of the metaproteomic sample indicated that the microbial community was similar to that from earlier metagenomic samples (Fig. 4, Supplementary Data 1).

The metaproteomic data obtained represented greater than 1% of the total number of proteins for seven MAGs (Supplementary Table 6), and the focus of the discussion will be on those. In this study, the proportion of genes identified as proteins is low, in comparison to another study using the same approach^33^, where about 9% of genes of 49 MAGs were identified as proteins. We attribute this difference to the need for autotrophy when H_2_ is the energy source. In contrast, when acetate is used as the energy source (as is the case in the other study^33^), it also serves as a carbon source, precluding the energy expenditure associated with CO_2_ fixation and allowing greater biomass build-up.

## Discussion

Data indicated that two MAGs, belonging to families Desulfobulbaceae (c16a) and Rhodospirillaceae (c57), were likely responsible for primary production in this ecosystem: proteomic results for the Desulfobulbaceae MAG confirmed its autotrophic potential by providing evidence for all the proteins in the reductive acetyl-CoA pathway (Fig. 6, Supplementary Table 7); the ability of the Rhodospirillaceae MAG to fix carbon via the Calvin cycle was also evidenced by its proteome (Supplementary Data 6). These two autotrophic microorganisms both utilize H_2_ as an electron donor, as they both harbour group 1 [NiFe]-hydrogenases (Supplementary Data 6), but they differ in their use of electron acceptors. The Desulfobulbaceae MAG is a sulfate-reducing bacterium and includes the entire dissimilatory sulfate-reducing pathway in its proteome (Fig. 6). In contrast, in the Rhodospirillaceae MAG, a dissimilatory sulfite reductase was identified in the proteome (Supplementary Data 6). Thus, this organism is likely to use intermediate valence sulfur species as terminal electron acceptors. Their presence can be explained by the introduction of O_2_ during previous samplings, which oxidized sulfide (S(-II)) or Fe(II) to Fe(III), which in turn oxidized S(-II). In the absence of this artefact, the abundance of Rhodospirillaceae is expected to be lower.

The five remaining microorganisms grow as heterotrophs in these conditions as surmised by the lack of detection of [NiFe]-type 1 hydrogenases from their proteome, while other proteins (for example, those pertaining to the acetyl-CoA pathway) are readily detected (Fig. 5, Supplementary Data 6)^34^. The *Hyphomonas* MAG (c22) is the only organism capable of degrading organic macromolecules, such as proteins and RNA. Based on the absence of a respiratory pathway and on the detection of proteins involved in acetate production, we propose that it ferments organic compounds derived from microbial necromass, producing acetate. Furthermore, the proteome of Peptococcaceae MAG c4a, an SRB, shows an abundance of aromatic compound degradation proteins (incomplete pathway of anaerobic toluene degradation), suggesting that it uses complex organic compounds derived from microbial necromass, in addition to ethanol, butyrate and formate, as electron donors and carbon sources (Supplementary Data 6). Three MAGs (c8a, c12 and c23) represent Gram-positive SRB with proteomic evidence supporting utilization of acetate and other organic acids and/or ethanol as electron donors (Supplementary Data 6). These last four heterotrophic SRB are expected to oxidize carbon to CO_2_ using the oxidative acetyl-CoA pathway (Supplementary Data 6).

From careful biochemical pathway annotation of the seven MAGs harbouring sufficient proteomic data, and from the geochemical background, we inferred a putative carbon cycle (Fig. 7, Supplementary Data 6). The two autotrophic organisms fix CO_2_ and produce biomass. The *Hyphomonas* MAG degrades microbial necromass and produces acetate. However, we observe no net accumulation of acetate in the water (Supplementary Fig. 6), presumably due to acetate oxidation to CO_2_ by the four heterotrophic SRB via the oxidative acetyl-CoA pathway. Other fermentation products (that is, ethanol, butyrate, formate) are also oxidized by these SRB, suggesting that the fermentation pathways of this system were not all identified.

Desulfobulbaceae MAG c16a appears to be adapted to a wide range of H_2_ concentrations. Indeed, this organism is abundant in the microbial community in the early anoxic phase, when H_2_ input, delivered through the gas permeable membrane, was only limited by gas/water equilibrium (Fig. 4, Supplementary Fig. 2). Later, between days 56 and 77, the H_2_ input decreased drastically (Supplementary Fig. 2). As a result, the overall concentration of planktonic cells decreased but, as shown by 16S rRNA sequencing, Desulfobulbaceae MAG c16a remained the most abundant microorganism. These data suggest that this organism is able to thrive at H_2_ concentrations ranging from saturated to non-detectable.

A limitation of deep subsurface microbial sampling is the possibility of contamination. In order to address this possibility, we collected 15 additional porewater samples from 7 other boreholes across the Mont-Terri Underground Rock Laboratory (Fig. 1) and carried out 16S rRNA analysis (Supplementary Data 7). By comparing the OTUs with the 16S rRNA gene derived from genomes, we could detect OTUs corresponding to the two autotrophic bacteria (Desulfobulbaceae and Rhodospirillaceae MAGs) and to MAG Peptococcaceae c8a in all tested boreholes (Supplementary Table 3), including that from borehole BHT-1 that was drilled under anoxic conditions using sterile precautions^35^. Thus, these three MAGs are surmised to be indigenous to Opalinus Clay rock. Average nucleotide analysis (Supplementary Table 5) of these three genomes revealed that they are distinct from known genomes, constituting new genera, which supports an Opalinus Clay origin. These findings suggest that autotrophic hydrogen-oxidizing bacteria are extant in undisturbed Opalinus Clay, without H_2_ amendment. In such conditions, these population could rely on two putative sources of H_2_ that are the radiolysis of water^7^,^24^ and fermentation pathways of autochthonous organic matter^5^,^24^.

SRB, which are the most abundant metabolic group in the microbial community characterized here, are known to accelerate the rate of steel corrosion under anoxic conditions^36^. At first glance, it may seem that the activity of SRB would have a negative impact on the safety of deep geological repository, by promoting the corrosion of one of the engineered barriers, whose primary function is to isolate radionuclides from the environment. However, careful design may harness the ability of microorganisms to consume H_2_ rapidly, reducing an overpressure that is otherwise expected in the repository^20^, while relegating sulfate reduction to an iron-rich porous medium that would sequester sulfide. For instance, by combining a barrier of bentonite, a swelling clay, around the canister with a higher permeability zone between the host rock and the bentonite, SRB may grow in the higher permeability zone where sulfide would precipitate, while relying on H_2_ diffusing from the canister surface across the bentonite. This mechanism can maintain the integrity of the host-rock as well as minimize the impact of sulfide on canister corrosion. Thus, microbial activity can be integrated into the design of nuclear waste disposal, and can have a positive impact on the long-term safety case of the repository.

Further investigations will require quantification of the rate of H_2_ consumption in this system. This is important in order to ascertain whether it is greater than the H_2_ production rate from anoxic steel corrosion. Furthermore, the rate of sulfate consumption is also of interest^37^, in order to determine whether it can be consumed faster than it can be replenished, via diffusion of porewater from Opalinus Clay. If sulfate can be depleted, methanogenic conditions could take hold. In that case, pressure in repositories might not decrease to the same extent, because methane is poorly soluble.

The present study provides detailed insight into the metabolic interactions of microorganisms in a subsurface ecosystem where the oxidation of H_2_ is the primary energy source. We are aware of the fact that it is based on conditions that are unrealistic for the undisturbed subsurface, due to the high concentrations of H_2_ delivered in this *in situ* experiment. However, such conditions enable biomass build-up and thus metaproteomic analysis, which has, to our knowledge, never been performed for microbial systems in the deep subsurface. This is a significant step forward because, unlike metagenomic analysis, which is based on the presence of genes and thus, can only describe potential metabolic activities, metaproteomic analysis ascertains the presence of proteins and thus, active metabolism. Used jointly, these methods are powerful. Metagenomic binning extracts individual MAGs from a complex microbial community, while metaproteomics describe the metabolic activity of these MAGs. Together, they can paint a detailed picture of an active metabolic network at the scale of a microbial community.

In this work, we describe a microbial system whose primary production is based on a chemolithoautrophic metabolism: a chemical reaction, H_2_ oxidation coupled with SO_4_^2−^ reduction, provides energy for microbial metabolism and for carbon fixation. The organic carbon generated is then available to the rest of the community, which can assimilate it for biomass build-up or oxidize it to gain energy. The consequence of the obvious lack of sunlight in deep subsurface environments is that carbon fixation depends on this type of metabolism. But this fact doesn't imply that this system is totally disconnected from sunlight. Indeed, unlike the model proposed by Pedersen^4^, the present one is based on the occurrence of sulfate (Fig. 7b). This means that it is ultimately connected to sunlight, because sulfate originates from sulfide oxidation on early Earth, after the onset of oxygenic photosynthesis^38^.

The proposed carbon cycling model may be extant in many deep subsurface environments. Indeed, this carbon cycling is expected to take place in the subsurface when H_2_ and SO_4_^2−^ are present concomitantly. H_2_ is a key metabolic compound for the deep subsurface^8^,^9^,^10^,^11^,^12^,^13^, and is produced *in situ* by serpentinization^6^ and by the radiolysis of water^7^. Despite the low level of H_2_ present in Opalinus Clay^24^, our work has suggested that autotrophic hydrogen-oxidizing SRB are extant in undisturbed Opalinus Clay, without H_2_ amendment. Thus, it is conceivable that carbon cycling takes place within this rock formation at exceedingly low rates in macropores, echoing the very slow microbial metabolism identified in deep ocean sediments^39^.

## Methods

### Detailed procedures

A complete description of the experimental procedures can be found in the Supplementary Information.

### Instrumented H_2_ injection borehole

A 25 m long borehole (borehole BRC-3) was drilled from the gallery floor. A hydraulic neoprene packer was installed at the bottom of the borehole, in order to create a 2.74 m long chamber isolated from oxic gallery atmosphere. Porewater, constantly produced by the borehole (at a rate of 20 ml per day), accumulated in the borehole. Multiple polyamide lines were placed for connecting this chamber to surface equipment, allowing water recirculation and sampling. To avoid lines clogging with particles, a polyvinyl chloride screen was also installed in the chamber. An artist's rendering of the borehole equipment is presented in Fig. 2.

The surface equipment, through which water borehole water was recirculated, consists of polyetheretherketone (PEEK) lines connected in a circulation loop to a plexiglas sediment trap, a peristaltic pump, a flow-meter, a dissolved oxygen probe, a gas permeable membrane connected to a 500 ml reservoir filled with 100% H_2_ and needle valves (Supplementary Fig. 7). In order to protect this experiment from oxygen contamination when borehole water was recirculated, a plexiglas cabinet was installed and regularly flushed with argon. Pure H_2_ was later directly and non-continuously injected into the borehole chamber, thus creating a gas phase. Sampling was carried out on a close to weekly basis. More details concerning experiment set-up of recirculation and non-recirculation modes can be found in Supplementary Fig. 7 and in the Supplementary Information. When a large volume of water was collected for molecular analyses (around 500 ml), it was replaced in the borehole by sterile and APW, whose composition is given in Supplementary Table 1.

### Chemical assays

To measure organic acids, samples were treated with BaCl_2_ and OnGuard Ag cartridges (Dionex) to remove the high level of sulfate and chloride, and the pH raised prior to their measurement by ion chromatography.

S(-II) was determined with Cline method, by reaction with *N*,*N*-Dimethyl-*p*-phenylenediamine. Absorbance was measured at 664 nm using a spectrophotometer^40^. Fe(II) were determined by reaction with ferrozine. Absorbance was measured at 562 nm using a spectrophotometer^41^.

Dissolved gases (H_2_ and CO_2_) were measured with a GC-FID (Agilent Technologies), using a headspace equilibration method.

### Planktonic cell density

Planktonic cell density was measured with SybrGreen I staining on an epifluorescence microscope. For the preparation, one ml of sample was filtered on a black polycarbonate membrane (mesh of 0.2 μm), rinsed three times with PBS, and finally stained and mounted with a solution of SyberGreen I (1:200 dilution) and polyvinylalcohol (moviol)^42^.

### DNA sampling and extraction

Water samples from the seven other boreholes were recovered in anoxic and sterile bottles, before filtration using sterile 0.2 μm polycarbonate filters. The filters were immediately placed in a 1.5 ml sterile tube containing 0.4 ml of LifeGuard Soil Preservation Solution (MO BIO Laboratories), prior to being frozen at −20 °C. The DNA of a first set of samples was extracted using a modified version of FastDNA SPIN Kit for Soil (MP Biomedicals) followed by a purification step using the standard protocol of the Genomic DNA Clean & Concentrator purification kit (Zymo Research).

Water samples from the H_2_ injection experiment were extracted using a second method that is phenol-chloroform extraction followed by an ethanol precipitation. The reason is that FastDNA SPIN Kit for Soil method recovered poor DNA quality (in term of fragment length) from samples containing S(-II) and black precipitates. It was decided to use a method that doesn't involve bead-beating. More details concerning DNA extraction protocols can be found in the Supplementary Information.

### 16S rRNA gene sequencing

Itag 16S rRNA sequencing was done by the Joint Genome Institute (Walnut Creek, CA) through a community sequencing program project (CSP 1,505). Libraries for Illumina MiSeq sequencing (a 2 × 250 bp reads configuration) were produced by amplifying region V4 of the 16S rRNA gene using primers 515F and 806R. Amplicons were then analysed using the JGI iTagger version 1.1 pipeline (bitbucket.org/berkeleylab/jgi_itagger/). However, the two last samples of H_2_ enrichment, which were recovered at days 483 and 505, were sequenced at the Research and Testing laboratory and were analysed by their own bioinformatic method. BLAST version 2.2.28 was then used to map OTUs of samples recovered at days 483 and 505 days to OTUs from the iTagger pipeline in order to merge all samples in a single biom file. In all 98.8% of Research and Testing laboratory reads could be mapped to iTaggers OTUs.

### Metagenomic sequencing and assembly

Samples recovered at days 181, 188, 195, 202, 206, 209, 214, 238, 246 and 250 were sequenced using Illumina HiSeq 2,500 at the Lausanne Genomic Technologies Facility. These 10 samples were co-assembled into contigs with Ray version 2.3.1 using a kmer length of 41. Samples recovered at days 14, 48, 101, 122, 134 and 233 were sequenced using Illumina HiSeq at the Joint Genome Institute.

### Contig binning

Bowtie 2 version 2.1.0 (http://bowtie-bio.sourceforge.net/bowtie2/) and MarkDuplicates from Picard tools version 1.77 (broadinstitute.github.io/picard/) were used to map quality trimmed reads from all 16 samples onto the contigs of the co-assembly, in order to calculate contig coverages across samples. Only contigs >5,000 bp were used as input for CONCOCT version 0.2 (ref. 43) (github.com/binpro/concoct). After binning, some bins were subdivided based on their coverage pattern across samples (Supplementary Data 2). Purity and completeness of each bin was then assessed using CheckM v.0.9.4 (ref. 31) (github.com/ecogenomics/checkm) using the lineage-specific workflow. Bins with completeness greater than 75% and a contamination smaller than 10% were considered as draft genomes that are high quality and nearly complete. Some bins were corrected manually, as explained in the Supplementary Methods.

### Taxonomic annotation of bins

Taxonomic annotations of MAGs were done by the ribosomal database project classifier version 2.7 (http://rdp.cme.msu.edu/) on 16S rRNA genes detected by Barrnap version 0.4.2 (http://www.vicbioinformatics.com/software.barrnap.shtml). If no 16S rRNA gene was detected, MLtreeMap version 2.061 (http://mltreemap.org/) annotations were used instead.

The ANI between the selected draft genomes and references genomes found in NCBI genome database (http://www.ncbi.nlm.nih.gov/genome/) was calculated using the default parameters of the Kostas Lab's ANI calculator (http://enve-omics.ce.gatech.edu/ani/).

### Pathway annotation of bins

Genes were annotated with the IMG pipeline ( img.jgi.doe.gov/). Metabolic pathways were manually annotated using KEGG (www.genome.jp/kegg/), MetaCyc (http://metacyc.org/) databases and textbook biochemical pathways^44^.

### Proportion of microorganisms in microbial community

The relative abundance of each bin greater than 500 kb in size was calculated using the coverage of each of its contigs.

### Metaproteomics

A single borehole water collected 483 days after the first H_2_ injection (0.9 l) was filtered by two Sterivex (EMD Millipore) 0.22 μm polyethersulfone membranes and directly frozen in dry ice. Cells were lysed in two steps. The frozen filters were cut into small pieces, pooled and immersed in sodium dodecyl sulfate lysis buffer^45^. Samples were then placed in a boiling water bath for 15 min^46^. Proteins were finally extracted by the trichloroacetic acid precipitation method. Peptides were subjected to 24 h multi-step chromatographic separation via the Ultimate 3,000 HPLC system connected to the mass spectrometer and measurements done using the Multi-Dimensional Protein Identification Technology (MuDPIT) approach. The peptide fragmentation was executed and recorded via an LTQ-Orbitrap-Elite mass spectrometer.

For protein identification, the raw spectra were searched against the protein database generated by groundwater sample sequencing (as described above), via Myrimatch v2.1 algorithm omictools.com/myrimatch-tool).

### Data availability

The raw 16S itag reads from JGI were deposited to NCBI SRA under accession number SRA244825.

The raw 16S reads from Research & Testing Laboratory were deposited to NCBI SRA under accession numbers SRX957365 and SRX957366.

16S rRNA gene sequences were deposited to NCBI GenBank under accession numbers KP901405-KP902411 and KP942780-KP942813.

The raw metagenomic reads from Lausanne Genomic Technologies Facility were deposited to NCBI SRA under accession numbers SRX951251, SRX955797, SRX955810, SRX955847, SRX957166, SRX957167, SRX957183, SRX957184, SRX957186 and SRX957187.

The raw metagenomic reads from JGI were deposited to NCBI SRA under accession numbers SRA246967, SRA246971, SRA246972, SRA246976, SRA246982 and SRA246984.

The 15 selected draft genomes were deposited in this way:

- Gammaproteobacteria bacterium BRH_c0: This Whole Genome Shotgun project has been deposited at DDBJ/EMBL/GenBank under the accession LADM00000000. The version described in this paper is version LADM01000000.
- Peptococcaceae bacterium BRH_c4a: This Whole Genome Shotgun project has been deposited at DDBJ/EMBL/GenBank under the accession LADN00000000. The version described in this paper is version LADN01000000.
- Peptococcaceae bacterium BRH_c4b: This Whole Genome Shotgun project has been deposited at DDBJ/EMBL/GenBank under the accession LADO00000000. The version described in this paper is version LADO01000000.
- Peptococcaceae bacterium BRH_c8a: This Whole Genome Shotgun project has been deposited at DDBJ/EMBL/GenBank under the accession LADP00000000. The version described in this paper is version LADP01000000.
- *Hoeflea* sp. BRH_c9: This Whole Genome Shotgun project has been deposited at DDBJ/EMBL/GenBank under the accession LADQ00000000. The version described in this paper is version LADQ01000000.
- *Desulfatitalea* sp. BRH_c12: This Whole Genome Shotgun project has been deposited at DDBJ/EMBL/GenBank under the accession LADR00000000. The version described in this paper is version LADR01000000.
- Desulfobulbaceae bacterium BRH_c16a: This Whole Genome Shotgun project has been deposited at DDBJ/EMBL/GenBank under the accession LADS00000000. The version described in this paper is version LADS01000000.
- Clostridiaceae bacterium BRH_c20a: This Whole Genome Shotgun project has been deposited at DDBJ/EMBL/GenBank under the accession LADT00000000. The version described in this paper is version LADT01000000.
- *Hyphomonas* sp. BRH_c22: This Whole Genome Shotgun project has been deposited at DDBJ/EMBL/GenBank under the accession LADU00000000. The version described in this paper is version LADU01000000.
- Peptococcaceae bacterium BRH_c23: This Whole Genome Shotgun project has been deposited at DDBJ/EMBL/GenBank under the accession LADV00000000. The version described in this paper is version LADV01000000.
- Hyphomonadaceae bacterium BRH_c29: This Whole Genome Shotgun project has been deposited at DDBJ/EMBL/GenBank under the accession LADW00000000. The version described in this paper is version LADW01000000.
- *Pseudomonas* sp. BRH_c35: This Whole Genome Shotgun project has been deposited at DDBJ/EMBL/GenBank under the accession LADX00000000. The version described in this paper is version LADX01000000.
- *Roseovarius* sp. BRH_c41: This Whole Genome Shotgun project has been deposited at DDBJ/EMBL/GenBank under the accession LADY00000000. The version described in this paper is version LADY01000000.
- Flavobacteriales bacterium BRH_c54: This Whole Genome Shotgun project has been deposited at DDBJ/EMBL/GenBank under the accession LADZ00000000. The version described in this paper is version LADZ01000000.
- Rhodospirillaceae bacterium BRH_c57: This Whole Genome Shotgun project has been deposited at DDBJ/EMBL/GenBank under the accession LAEA00000000. The version described in this paper is version LAEA01000000.

All the other contigs (unclassified) were deposited in this way:

- Rock porewater metagenome: This Whole Genome Shotgun project has been deposited at DDBJ/EMBL/GenBank under the accession LADL00000000. The version described in this paper is version LADL01000000.

Gene annotation is available at IMG JGI portal, under Taxon ID 3300002468.

Additional data that support the findings of this study are available within the Supplementary Information files.

## Additional information

**How to cite this article:** Bagnoud, A *et al*. Reconstructing a hydrogen-driven microbial metabolic network in Opalinus Clay rock. *Nat. Commun.* 7:12770 doi: 10.1038/ncomms12770 (2016).

## Supplementary Material

Supplementary Information Supplementary Figures 1-7, Supplementary Tables 1-7, Supplementary Methods and Supplementary References

Supplementary Data 1 Number of OTUs observation for every sample. The last column gives a taxonomic affiliation for each OTU. The sequences of the two last samples (day_482 and day_505) were obtained from a different sequencing facility, but were mapped on OTUs defined for the 31 other samples. OTUs 1065 to 1098 corresponds to OTUs only present in these two last samples (day_482 and day_505). More information is available in the Supplementary Methods.

Supplementary Data 2 Binning table indicating the raw CONCOCT output and the same output that was manually corrected

Supplementary Data 3 Protein profile of sample recovered at day 483. Average adjusted protein NSAF was calculated by averaging the NSAF of run 1 (Supplementary Data 4) and run 2 (Supplementary Data 5) and then by multiplying this value by 10^5^.

Supplementary Data 4 Protein profile of the first run of the sample recovered at day 483. Adjusted NSAF were calculated by multiplying NSAF by 10^5^.

Supplementary Data 5 Protein profile of the second run of sample recovered at day 483. Adjusted NSAF were calculated by multiplying NSAF by 10^5^.

Supplementary Data 6 List of proteins identified Desulfobulbaceae c16a. For an exhaustive list of proteins measured, refer to Supplementary Data 3.

Supplementary Data 7 Number of observation of all 612 OTUs in 15 samples from 7 other boreholes. OTUs are identical to the ones of Supplementary Data 1.
