# Supplementary material for: Reconstructing a hydrogen-driven microbial metabolic network in Opalinus Clay rock
Source: Nat Commun. 2016 Oct 14;7:12770. doi: 10.1038/ncomms12770 (PMC5067608; doi:10.1038/ncomms12770)
Supplement: Supplementary Information — Supplementary Figures 1-7, Supplementary Tables 1-7, Supplementary Methods and Supplementary References [file ncomms12770-s1.pdf]

SUPPLEMENTARY FIGURES

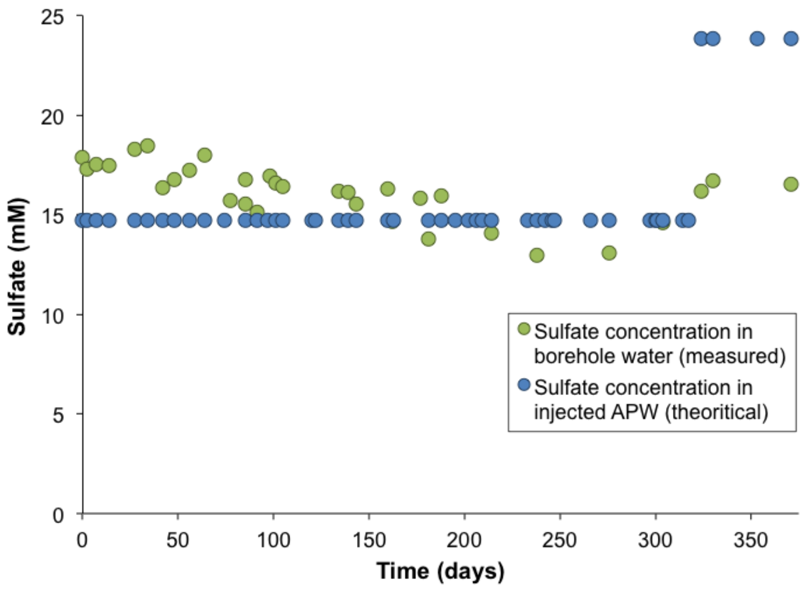

Supplementary Figure 1. Measured sulfate concentration of borehole water (green dots) and theoretical concentration of sulfate in artificial porewater (APW) injected in the course of the experiment (blue dots). Till day 317, APW I containing 14.7 mM was injected. Later, starting day 324, APW II containing 23.83 mM of sulfate was injected instead, to better match the natural sulfate concentration of the water produced by borehole BRC-3. During the first phase when APW I was injected, the decreasing sulfate concentrations are mainly due to dilution of borehole water in APW I than contains lower amount of sulfate. However, the fact that sulfate concentrations in borehole get lower than in APW I after 200 days indicates that sulfate is consumed by another process, which is thought to be sulfate reduction.

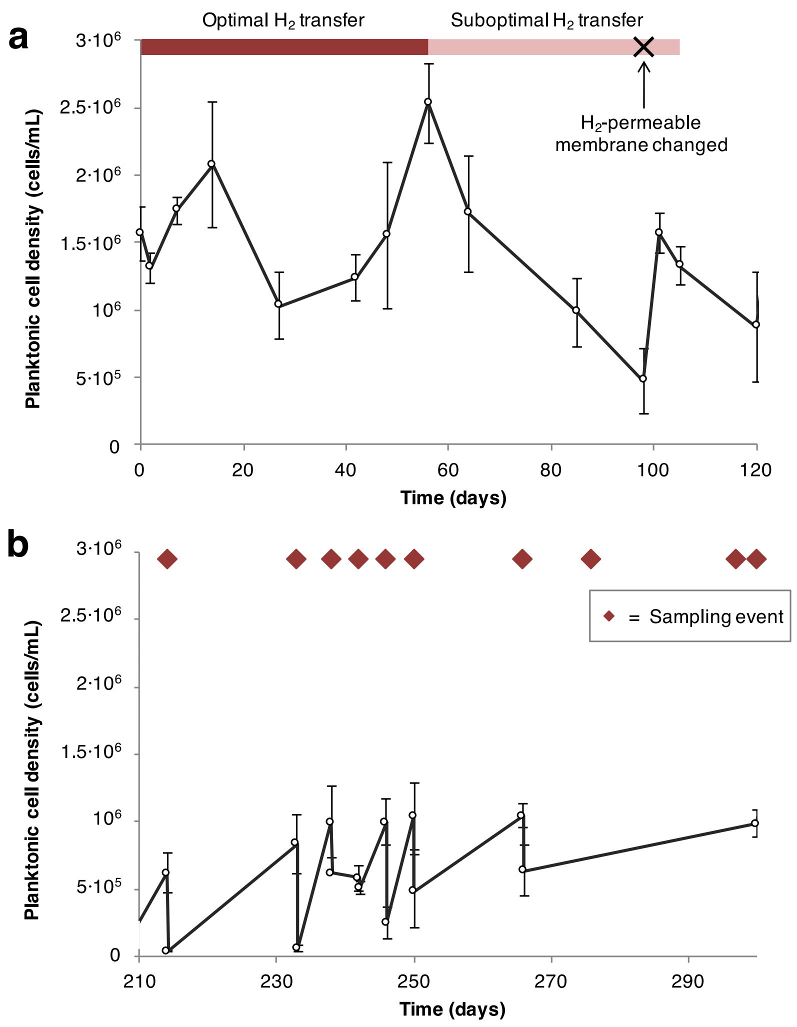

Supplementary Figure 2. H_2 consumption in the borehole over time (a) during the phase when water was recirculated and H_2 continuously injected and (b) during the phase when water was not recirculated and H_2 delivered through discrete injections into the borehole. (a) H_2 concentration was at saturation immediately past the gas permeable membrane (green line and open circles), but almost null in the water coming back up from the borehole (blue line and open circles). This clearly shows H_2 consumption in the borehole. Between 56 and 77 days, the gas permeable membrane became clogged. It is the reason it was decided to inject H_2 directly into the borehole, thus creating a gas phase. (b) Each increase in H_2 concentration is due to an injection during a sampling event (red diamonds), and each decrease highlights H_2 consumption in the borehole during the sulfate reduction phase (Fig. 3).

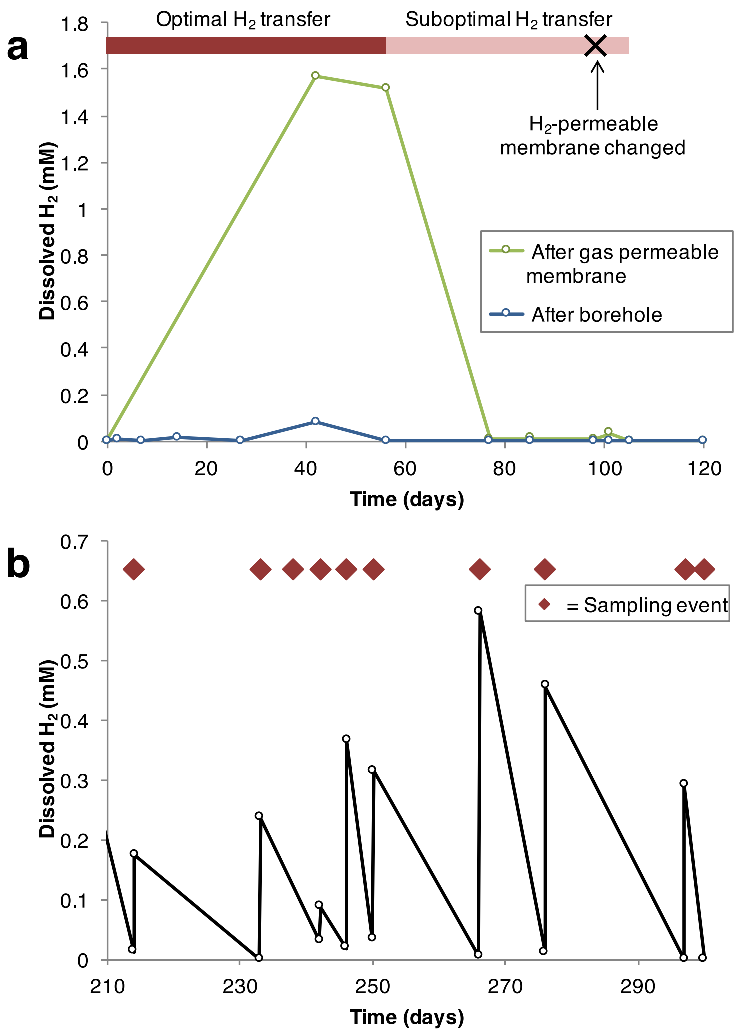

Supplementary Figure 3. Planktonic cell density (black lines and open circles) over time in borehole water (a) during the phase when water was recirculated and H_2 continuously injected (b) during the phase when water was not recirculated and H_2 delivered through discrete injections into the borehole. (a) The sample at day 0 was recovered prior to any H_2 amendment. The first planktonic cell density peak (at day 14) corresponds to the suboxic phase, when O_2 is still present in borehole, and when the Xanthomonadaceae and *Pseudomonas* populations are at their maximum (Fig. 4). After 27 days, O_2 concentration dropped to zero (Fig. 3), causing the decrease of suboxic microorganisms. At 56 days, a second peak coincides with the maximal Fe(II) concentration measured (Fig. 3), and highlights the growth of anaerobic microorganisms. However, after 56 days, H_2 transferred stopped suddenly (Supplementary Fig. 2), impacting microbial growth. A third peak appears immediately after replacing the H_2-permeable membrane (black cross at 98 days) but because it was clogged again soon thereafter, planktonic cell density rapidly dropped. (b) For each sampling event (red diamonds), sampled borehole water was replaced with synthetic porewater (that was sterile and anoxic). This is why every second measurement of planktonic cell density is lower, because it was carried out after having replaced the borehole water. But after a few days, planktonic cell density always increased (except one time, between 238 and 242 days), indicating that microorganisms were growing during sulfate reduction phase (Fig. 3). Planktonic cell density is missing for the sampling event of day 276 because, unlike all other samples that were measured right after they were recovered, the samples were stored two days at 4 ˚C, leading to biased results.

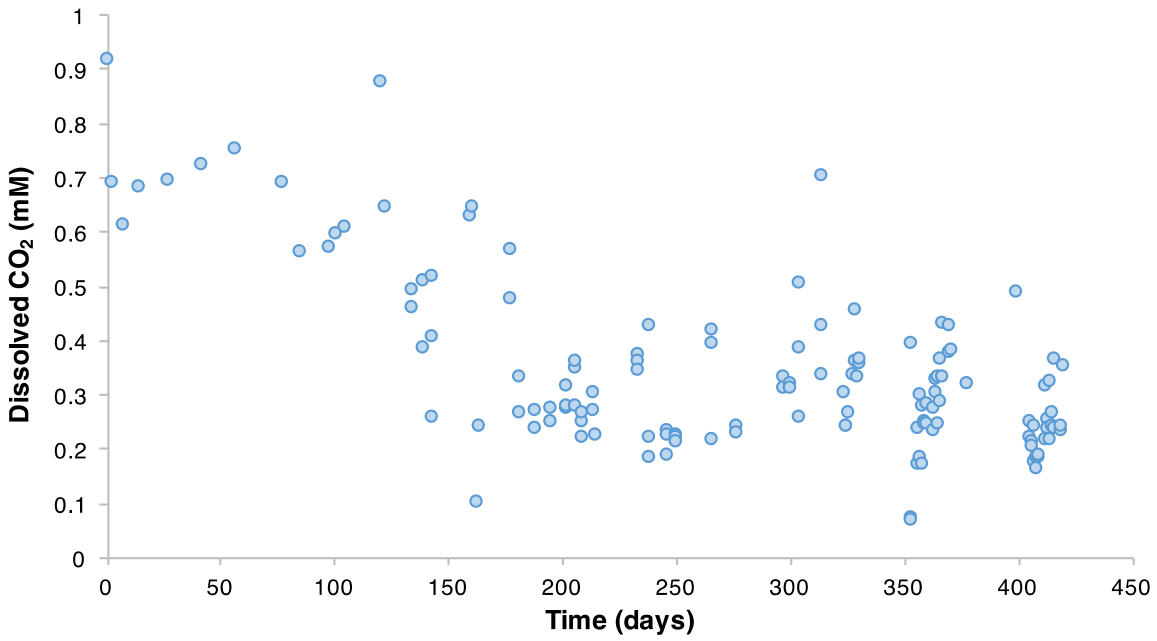

Supplementary Figure 4. CO_2 concentration over time in borehole water. This decrease indicates that CO_2 is consumed by autotrophic microorganisms.

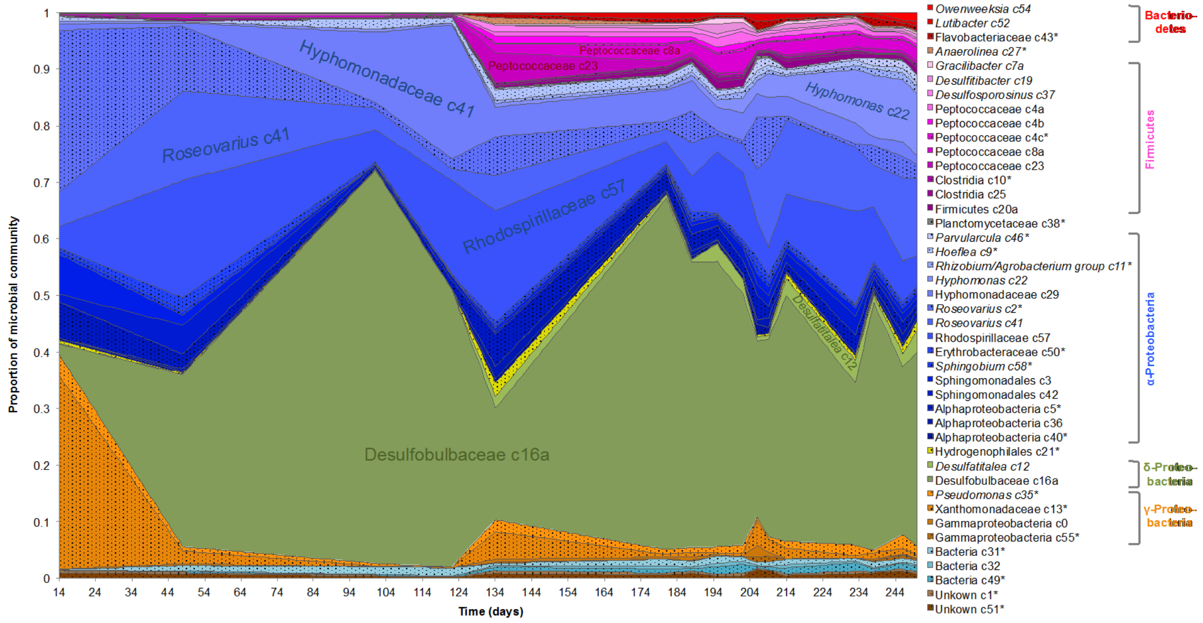

Supplementary Figure 5. Microbial community composition over time, based on the taxonomic affiliation of genomes obtained with metagenomic analysis. The dashed area and bins labeled with * correspond to bins that are not high-quality enough to be considered as draft genomes. See Supplementary Tables 2 and 4 for more information.

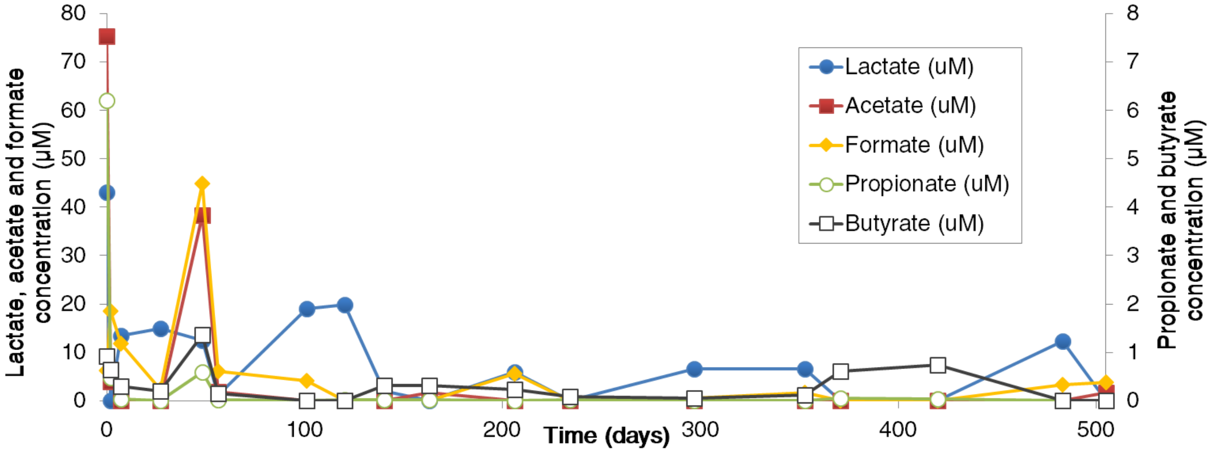

Supplementary Figure 6. Organic acid concentration over time in borehole water: lactate (solid blue circles), acetate (solid red squares), formate (solid yellow diamonds), propionate (open green circles) and butyrate (open black squares). Solid symbols belong to the left Y-axis, while open symbols belong to the right Y-axis.

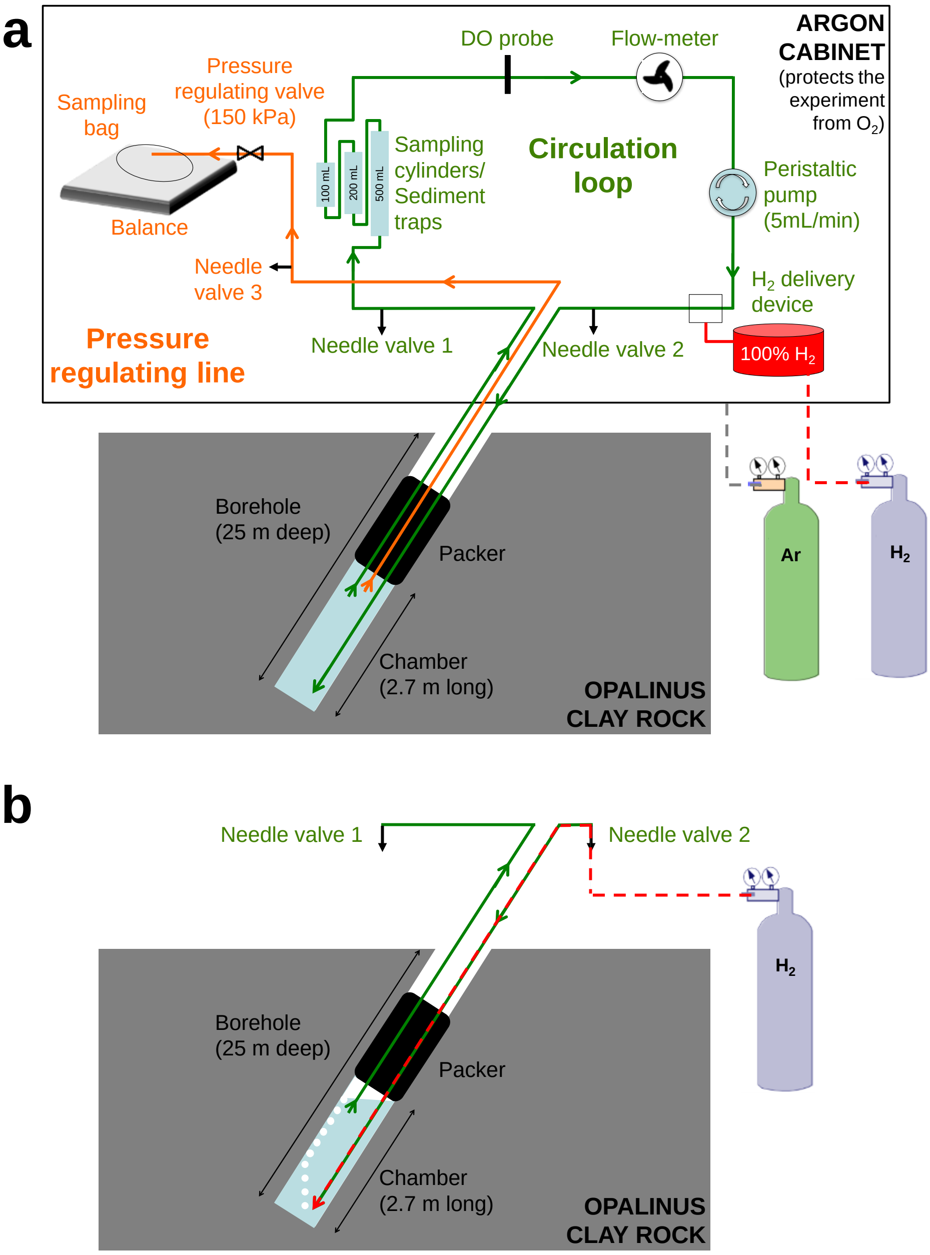

Supplementary Figure 7. Experimental set-up: (a) in recirculation mode with continuous H2 injection and (b) in non-recirculation mode with discrete H2 injections into the borehole. Dashed lines represent temporary connections between gas tanks and elements of the experiment. (a) In circulation mode, water is withdrawn from the borehole by the upper green line and goes through a first needle valve (needle valve 1), 5 cylinders that act as sediment traps (only three are indicated in the cartoon), a DO probe, a peristaltic pump (5 mL/min), a flow-meter, a gas-permeable membrane in contact with a H2 reservoir, and a second needle-valve (needle valve 2), before being re-injected into the bottom of the borehole. The third line (in orange) served as a pressure valve, in order to keep a stable pressure of 0.5 relative bars in the borehole. Additional water produced by the borehole was removed and collected into a bag. Here, surface equipment is kept under anoxic conditions in an argon-flushed cabinet in order to prevent O2 from contaminating borehole water. (b) In non-recirculation mode, only needle valves are used for borehole water sampling, artificial water injection (not shown in the cartoon) and H2 injection that created a gas phase at the top of borehole chamber.

SUPPLEMENTARY TABLES

Supplementary Table 1. Natural BRC-3 borehole water and artificial porewater (APW) composition used for replacing sampled borehole water. APW1 was used from day 0 till day 317. Later only APW II was used. 'n.d.' stands for not determined.

	Na^+ (mM)	Mg^{2+} (mM)	K^+ (mM)	Ca^{2+} (mM)	Cl^- (mM)	SO_4^{2-} (mM)	HCO_3^- (mM)
Artificial porewater I	255.59	17.89	1.46	15.79	294.58	14.70	0.57
Artificial porewater II	270.00	23.02	2.12	18.88	307.76	23.83	0.57

Supplementary Table 2. Completeness and contamination of bins assessed by the lineage mode of CheckM. Bins with completeness greater than 75% and with a contamination smaller than 10% were considered as MAGs. These bins are highlighted by the black frame. Also indicated in this table for each bin is its number of contigs, its size (total contigs length) and its average proportion in the microbial community (only for bins larger than 500 kb).

Bin	Number of contigs	Bin size (Mbp)	Proportion (for bins > 0.5 Mbp)	Completeness	Contamination
c16a	72	6.52	40.5%	100%	1%
c8a	48	4.17	1.5%	100%	2%
c41	71	5.01	7.9%	99%	8%
c12	91	6.00	1.4%	99%	3%
c20a	92	4.51	0.6%	98%	5%
c4a	98	3.49	0.6%	98%	1%
c4b	66	3.62	0.4%	98%	1%
c32	43	3.49	0.2%	98%	2%
c19	48	3.85	0.4%	98%	4%
c29	96	3.70	6.3%	97%	2%
c22	140	4.07	3.6%	97%	7%
c7a	98	4.11	0.2%	96%	3%
c54	69	2.92	0.2%	95%	2%
c23	348	6.03	0.5%	95%	4%
c37	313	6.23	0.3%	94%	4%
c25	257	5.20	0.2%	94%	7%
c49	193	6.40	0.6%	94%	98%
c52	84	3.18	0.6%	92%	7%
c0	140	3.53	0.3%	90%	1%
c36	236	3.72	0.5%	81%	2%
c42	147	2.19	1.6%	81%	1%
c3	157	2.51	1.3%	80%	1%
c57	274	3.91	9.7%	78%	1%
c35	296	3.14	1.2%	72%	10%
c58	378	4.33	0.5%	71%	4%
c40	213	2.82	0.5%	69%	1%
c11	281	3.49	0.5%	67%	1%
c9	279	3.86	1.0%	65%	1%
c5	244	2.79	2.5%	59%	0%
c27	258	2.28	0.2%	56%	0%
c50	143	1.53	0.6%	55%	0%
c13	187	1.94	4.0%	53%	1%
c46	191	2.19	0.5%	52%	1%

Bin	Number of contigs	Bin size (Mbp)	Proportion (for bins > 0.5 Mbp)	Completeness	Contamination
c43	193	2.51	0.4%	46%	2%
c4c	254	2.94	0.2%	40%	1%
c2	143	1.58	5.5%	37%	0%
c21	140	1.04	0.5%	24%	1%
c38	232	1.71	0.1%	20%	1%
c55	109	0.87	0.2%	16%	0%
c10	85	0.64	0.1%	14%	0%
c34	46	0.32	-	9%	0%
c20b	7	0.05	-	9%	0%
c44	10	0.07	-	7%	0%
c18	44	0.30	-	7%	0%
c47	46	0.33	-	5%	0%
c1	56	0.54	0.3%	4%	0%
c30	35	0.24	-	3%	0%
c24	49	0.43	-	2%	0%
c14	28	0.27	-	0%	0%
c15	22	0.15	-	0%	0%
c16b	5	0.16	-	0%	0%
c17	1	0.01	-	0%	0%
c26	46	0.34	-	0%	0%
c28	1	0.01	-	0%	0%
c31	52	0.71	0.9%	0%	0%
c33	7	0.04	-	0%	0%
c39	25	0.19	-	0%	0%
c45	7	0.05	-	0%	0%
c48	7	0.06	-	0%	0%
c51	118	1.20	0.8%	0%	0%
c56	6	0.05	-	0%	0%
c6	3	0.02	-	0%	0%
c7b	22	0.18	-	0%	0%
c8b	4	0.03	-	0%	0%
c8c	2	0.01	-	0%	0%

Supplementary Table 3. Analysis of 16S rRNA genes recovered from metagenomic binning. The table includes 16S RNA gene localization and length, its RDP classification, its NCBI BLASTN best hit, and its corresponding OTU number from the 16S rRNA sequencing project encompassing 23 samples coming from 8 boreholes (Supplementary Data 7). MAGs are shaded in grey.

							NCBI BLASTN			Comparison with itag sequencing (region V4 of 16S rRNA gene)			
Bin	Contig ID	Gene start position	Gene stop position	Gene length (bp)	Is 16S rRNA gene complete ?	RDP taxonomic affiliation	Best hit	Reference	Identity	OTU #	Alignment length	Alignement identity	OTU occurence in other boreholes
c2	BRHa_1000274	531	1996	1466	yes	*Roseovarius*	*Roseovarius tolerans*	NR_026405.1	96%		*no match found*		
c4a	BRHa_1002702	2	494	493	no	Peptococcaceae	*Desulfotomaculum gibsoniae*	NR_114759.1	92%		*no match found*		
c4b	BRHa_1004082	38170	39701	1531	yes	Peptococcaceae	*Cryptanaerobacter phenolicus*	NR_025757.1	90%	29	253	100	5/7
	BRHa_1000636	1	324	324	no	Peptococcaceae	*terephthalicicum*	.1	88%		*no match found*		
c5	BRHa_1000961	18676	20170	1495	yes	Alphaproteobacteria	*Pannonibacter phragmitetus*	NR_028009.1	89%	11	253	100	5/7
c7a	BRHa_1002652	3979	5498	1520	yes	*Gracilibacter*	*Gracilibacter thermotolerans*	.1	97%	70	253	100	3/7
c8a	BRHa_1005448	227403	227813	411	no	Peptococcaceae	*Desulfotomaculum gibsoniae*	NR_103939.1	92%		*no match found*		
	BRHa_1000843	1	559	559	no	Peptococcaceae	*Desulfotomaculum gibsoniae*	NR_103939.1	93%		*no match found*		
	BRHa_1004053	339	1868	1530	yes	Peptococcaceae	*Desulfotomaculum thermosapovorans*	NR_044948.1	93%	7	253	100	7/7
	BRHa_1004053	5834	7322	1489	no	Peptococcaceae	*Desulfotomaculum gibsoniae*	NR_114761.1	93%	7	253	100	7/7
c9	BRHa_1003556	1	1000	1000	no	*Hoeflea*	*Hoeflea marina*	NR_043007.1	99%		*no match found*		
c11	BRHa_1003273	4602	6083	1482	yes	*Rhizobium*	*Rhizobium selenitireducens*	NR_044216.1	99%	65	253	100	3/7
c12	BRHa_1002295	424014	425575	1562	yes	*Desulfatitalea*	*Desulfatitalea tepidiphila*	NR_113315.1	97%	46	253	100	3/7
c13	BRHa_1004451	10345	11881	1537	yes	Xanthomonadaceae	*Rehaibacterium terrae*	NR_118587.1	95%	4	253	100	7/7
c15	BRHa_1001180	2116	3573	1458	yes	*Brevundimonas*	*Brevundimonas intermedia*	NR_041966.1	99%	48	253	100	6/7
	BRHa_1000106	2	1385	1384	no	*Desulfonispora*	*Desulfonispora thiosulfatigenes*	NR_026497.1	93%	24	253	100	6/7
c16a	BRHa_1003205	4192	5733	1542	yes	Desulfobulbaceae	*Desulfobacterium catecholicum*	NR_028895.1	95%	0	253	100	7/7
	BRHa_1002043	112079	112441	363	no	*Desulforhopalus*	*Desulforhopalus vacuolatus*	NR_044653.1	93%		*no match found*		
	BRHa_1000294	1	348	348	no	*Desulforhopalus*	*Desulforhopalus vacuolatus*	NR_044653.1	93%		*no match found*		
c19	BRHa_1004424	73008	73383	376	no	*Desulfitibacter*	*Desulfitibacter alkalitolerans*	NR_042962.1	95%		*no match found*		
	BRHa_1005141	47219	47602	384	no	*Desulfitibacter*	*Desulfitibacter alkalitolerans*	NR_042962.1	95%		*no match found*		
c20a	BRHa_1004490	145	1673	1529	yes	Firmicutes	*Proteiniborus ethanoligenes*	NR_044093.1	85%	26	253	100	2/7
c22	BRHa_1004732	3751	5202	1452	yes	*Hyphomonas*	*Hyphomonas neptunium*	NR_074092.1	97%	34	253	100	5/7
c24	BRHa_1000784	317	1848	1532	yes	*Coraliomargarita*	*Coraliomargarita akajimensis*	NR_074901.1	86%	49	253	100	2/7
c27	BRHa_1007068	9652	10007	356	no	*Anaerolinea*	*Anaerolinea thermophila*	NR_074383.1	87%		*no match found*		
c28	BRHa_1004480	3294	4831	1538	yes	*Escherichia/Shigella*	*Escherichia fergusonii*	NR_074902.1	99%	590	253	100	1/7
c29	BRHa_1007353	12	780	769	no	*Hyphomonas*	*Hyphomonas adhaerens*	NR_024937.1	99%	12	253	100	6/7
c32	BRHa_1006978	36856	37389	534	no	Bacteria	*Ignavibacterium album*	NR_074698.1	86%		*no match found*		
c35	BRHa_1000755	3721	5253	1533	yes	*Pseudomonas*	*Pseudomonas chloritidismutans*	NR_115115.1	99%	2	253	100	7/7
c37	BRHa_1002890	2	377	376	no	Desulfosporosinus	*Desulfosporosinus lacus*	NR_042202.1	96%		*no match found*		
	BRHa_1004950	1	333	333	no	Desulfosporosinus	*Desulfosporosinus lacus*	NR_042202.1	96%		*no match found*		
	BRHa_1004477	1	365	365	no	Desulfosporosinus	*Desulfosporosinus lacus*	NR_042202.1	97%		*no match found*		
c46	BRHa_1000455	2895	4356	1462	yes	*Parvularcula*	*Parvularcula oceani*	NR_125641.1	92%	60	253	100	3/7
c49	BRHa_1003600	36114	36476	363	no	Bacteria	*Caloramator indicus*	NR_026134.1	83%		*no match found*		
	BRHa_1002025	2	324	323	no	Bacteria	*Caloramator indicus*	NR_026134.1	81%		*no match found*		
c52	18	120	1631	1512	yes	*Lutibacter*	*Lutibacter maritimus*	NR_116738.1	95%	42	253	99.6	5/7
c54	BRHa_1002744	1	1328	1328	no	*Owenweeksia*	*Owenweeksia hongkongensis*	.1	89%	188	253	100	0/7
c57	BRHa_1007414	585	2069	1485	yes	Rhodospirillaceae	*Marispirillum indicum*	NR_044545.1	94%	1	253	100	7/7
c58	BRHa_1003529	3616	5101	1486	yes	*Sphingobium*	*Sphingobium xenophagum*	NR_026304.1	99%	89	253	99.21	6/7

Supplementary Table 4. Taxonomic affiliation of all bins, based on 16S rRNA gene sequence or, if missing, on MLTreeMap annotation. See Supplementary Methods for more detailed information concerning taxonomic annotation. MAGs are shaded in grey. For those, ANI analysis was carried out (See Supplementary Table 5 for more information). IMG phylogenetic distribution is also indicated for each bin, even though this information was not used for assessing the taxonomic affiliation of bins.

	16S rRNA gene			MLtreeMap taxonomic annotation			
Bin	RDP taxonomic affiliation	NCBI BLASTN best hit	Average Nucleotide Identity	using default tree	using geba tree	IMG phylogenetic distribut	Final annotation
c0			Haliea salexigens (77%)	Saccharophagus (71%)	Gammaproteobacteria (100%)	Gammaproteobacteria (69%)	Gammaproteobacteria
c1						Pseudomonas (55%)	
c2	Roseovarius	Roseovarius tolerans (96%)		Rhodobacteraceae (100%)	Rhodobacteraceae (100%)	Rhodobacteraceae (85%)	Roseovarius
c3			Erythrobacter citreus (81%)	Erythrobacter (94%)	Sphingomonadales (100%)	Sphingomonadales (85%)	Sphingomonadales
c4a	Peptococcaceae	Desulfotomaculum gibsoniae (92%)	Desulfotomaculum gibsoniae (79%)	Peptococcaceae (100%)	Clostridia (100%)	Peptococcaceae (71%)	Peptococcaceae
c4b	Peptococcaceae<br>Peptococcaceae	Cryptanaerobacter phenolicus (90%)<br>Pelotomaculum terephthalicicum (88%)	Desulfotomaculum gibsoniae (82%)	Peptococcaceae (100%)	Peptococcaceae (100%)	Peptococcaceae (74%)	Peptococcaceae
c4c				Peptococcaceae (100%)	Peptococcaceae (89%)	Peptococcaceae (62%)	Peptococcaceae
c5	Alphaproteobacteria	Pannonibacter phragmitetus (89%)		Rhodospirillales (100%)	Alphaproteobacteria (100%)	Alphaproteobacteria (74%)	Alphaproteobacteria
c6						Bacteroidales (78%)	
c7a	Gracilibacter	Gracilibacter thermotolerans (97%)	No genome match	Desulfitobacterium (83%)	Desulfitobacterium (83%)	Peptococcaceae (69%)	Gracilibacter
c7b						Clostridiales (45%)	
c8a	Peptococcaceae<br>Peptococcaceae<br>Peptococcaceae<br>Peptococcaceae	Desulfotomaculum gibsoniae (92%)<br>Desulfotomaculum gibsoniae (93%)<br>Desulfotomaculum thermosapovorans (93%)<br>Desulfotomaculum gibsoniae (93%)	Desulfotomaculum alcoholivorax (78%)	Peptococcaceae (100%)	Peptococcaceae (100%)	Desulfotomaculum (68%)	Peptococcaceae
c8b						Desulfosporosinus (82%)	
c8c						Desulfosporosinus (100%)	
c9	Hoeflea	Hoeflea marina (99%)	Hoeflea sp. (83%)	Rhizobiales (100%)	Rhizobiales (92%)	Hoeflea (52 %)	Hoeflea
c10				Clostridia (67%)	Peptococcaceae (67%)	Clostridia (51%)	Clostridia
c11	Rhizobium	Rhizobium selenitireducens (99%)		Rhizobiaceae (100%)	Rhizobium (100%)	Rhizobiaceae (80%)	Rhizobium
c12	Desulfatitalea	Desulfatitalea tepidiphila (97%)	Desulfococcus multivorans (77%)	Deltaproteobacteria (79%)	Desulfococcus (93%)	Desulfobacteraceae (49%)	Desulfatitalea
c13	Xanthomonadaceae	Rehaibacterium terrae (95%)		Xanthomonadaceae (100%)	Xanthomonadaceae (100%)	Xanthomonadaceae (58%)	Xanthomonadaceae
c14						Alphaproteobacteria (78%)	
c15	Brevundimonas<br>Desulfonispora	Brevundimonas intermedia (99%)<br>Desulfonispora thiosulfatigenes (93%)				Alphaproteobacteria (75%)	
c16a	Desulfobulbaceae<br>Desulforhopalus<br>Desulforhopalus	Desulfobacterium catecholicum (95%)<br>Desulforhopalus vacuolatus (93%)<br>Desulforhopalus vacuolatus (93%)	Desulfocapsa sulfexigens (76%)	Desulfotalea (79%)	Desulfotalea (79%)	Desulfobulbaceae (42%)	Desulfobulbaceae
c16b						Alphaproteobacteria (77%)	
c17						Pseudomonas (86%)	
c18				Enterobacteriaceae (100%)	Shigella (100%)	Escherichia coli (87%)	Enterobacteriaceae
c19	Desulfitibacter<br>Desulfitibacter	Desulfitibacter alkalitolerans (95%)<br>Desulfitibacter alkalitolerans (95%)	Desulfitibacter alkalitolerans (78%)	Clostridiales (80%)	Clostridia (80%)	Peptococcaceae (34%)	Desulfitibacter
c20a	Firmicutes	Proteiniborus ethanoligenes (85%)	No genome match	Clostridia (100%)	Clostridiales (70%)	Clostridiales (64%)	Firmicutes
c20b				Moorella (100%)	Moorella (100%)	Clostridia (72%)	Clostridia
c21				Thiobacillus (100%)	Thiobacillus (100%)	Thiobacillus (71%)	Hydrogenophilales
c22	Hyphomonas	Hyphomonas neptunium (97%)	Hyphomonas oceanitis (83%)	Hyphomonadaceae (100%)	Hyphomonadaceae (100%)	Hyphomonas (54%)	Hyphomonas
c23			Desulfotomaculum gibsoniae (81%)	Desulfitobacterium (100%)	Desulfitobacterium (100%)	Desulfosporosinus (63%)	Peptococcaceae
c24	Coraliomargarita	Coraliomargarita akajimensis (86%)		Chlamydiales (100%)	Opitutus (100%)	Alphaproteobacteria (43%)	Coraliomargarita
c25			No genome match	Clostridia (94%)	Clostridiaceae (88%)	Clostridiales (63%)	Clostridia
c26						Alphaproteobacteria (67%)	
c27	Anaerolinea	Anaerolinea thermophila (87%)		Chloroflexi (78%)	Chloroflexi (78%)	Anaerolinea (46%)	Anaerolinea
c28	Escherichia/Shigella	Escherichia fergusonii (99%)					Escherichia/Shigella
c29	Hyphomonas	Hyphomonas adhaerens (99%)	Hyphomonas sp. (83%)	Hyphomonas (100%)	Hyphomonas (100%)	Hyphomonadaceae (73%)	Hyphomonas
c30				Gramella (100%)	Flavobacteriaceae (100%)	Flavobacteriaceae (73%)	Flavobacteriaceae
c31				Bacteria (100%)	Bacteria (100%)	Deltaproteobacteria (53%)	Bacteria
c32	Bacteria	Ignavibacterium album (86%)	Ignavibacterium album (70%)	Bacteroidetes/Chlorobi group (100%)	Bacteroidetes/Chlorobi group (100%)	Ignavibacteriaceae (67%)	Bacteria
c33						Bacteria (95%)	
c34				Alteromonadaceae (100%)	Gammaproteobacteria (100%)	Gammaproteobacteria (73%)	Gammaproteobacteria
c35	Pseudomonas	Pseudomonas chloritidismutans (99%)	Pseudomonas xanthomarina (98%)	Pseudomonas (100%)	Pseudomonas (100%)	Pseudomonas (93%)	Pseudomonas
c36			Candidatus Filomicrobium marinum (76%)	Alphaproteobacteria (100%)	Alphaproteobacteria (100%)	Rhizobiales (71%)	Alphaproteobacteria
c37	Desulfosporosinus<br>Desulfosporosinus<br>Desulfosporosinus	Desulfosporosinus lacus (96%)<br>Desulfosporosinus lacus (97%)<br>Desulfosporosinus lacus (97%)		Desulfitobacterium (100%)	Desulfitobacterium (100%)	Desulfosporosinus (61%)	Desulfosporosinus
c38				Rhodopirellula (83%)	Rhodopirellula (100%)	Planctomyces (82%)	Planctomycetes
c39						Alphaproteobacteria (67%)	
c40				Alphaproteobacteria (95%)	Alphaproteobacteria (100%)	Alphaproteobacteria (77%)	Alphaproteobacteria
c40x*				Sphingomonadales (100%)	Sphingomonadales (100%)	Sphingomonadales (71%)	Sphingomonadales
c41	Roseovarius	Roseovarius mucosus (99%)	Roseovarius sp. (98%)	Rhodobacteraceae (100%)	Rhodobacteraceae (100%)	Roseovarius (84%)	Roseovarius
c42			Porphyrobacter cryptus (78%)	Sphingomonadales (100%)	Sphingomonadales (100%)	Sphingomonadales (80%)	Sphingomonadales
c43				Flavobacteriaceae (100%)	Flavobacteriaceae (100%)	Flavobacteriaceae (77%)	Flavobacteriaceae
c44					Aquificales (50%)	Alphaproteobacteria (36%)	
c45						Pseudomonas (56%)	
c46	Parvularcula	Parvularcula oceani (92%)		Alphaproteobacteria (98%)	Alphaproteobacteria (100%)	Alphaproteobacteria (73%)	Parvularcula
c47				Bifidobacterium (100%)	Beutenbergia (100%)	Actinomycetales (76%)	Bacteria
c48						Alphaproteobacteria (64%)	
c49	Bacteria<br>Bacteria	Caloramator indicus (83%)<br>Caloramator indicus (81%)		Clostridia (70%)	Firmicutes (90%)	Clostridia (56%)	Bacteria
c50				Erythrobacter (95%)	Erythrobacter (100%)	Erythrobacteraceae (75%)	Erythrobacteraceae
c51						Rhodobacteraceae (56%)	
c52	Lutibacter	Lutibacter maritimus (95%)	Tenacibaculum maritimum (77%)	Flavobacteriaceae (100%)	Flavobacteriaceae (100%)	Flavobacteriacea (80%)	Lutibacter
c53*	Sphingobium	Sphingobium xenophagum (99%)		Sphingomonadales (100%)	Sphingomonadaceae (100%)	Sphingomonadales (70%)	Sphingobium
c54	Owenweeksia	Owenweeksia hongkongensis (89%)	Crocinitomix catalasitica (73%)	Bacteroidetes (100%)	Bacteroidetes (100%)	Bacteroidetes (89%)	Owenweeksia
c55				Gammaproteobacteria (67%)	Gammaproteobacteria (67%)	Gammaproteobacteria (51%)	Gammaproteobacteria
c56						Proteobacteria (75%)	
c57	Rhodospirillaceae	Marispirillum indicum (94%)	Caenispirillum salinarum (77%)	Rhodospirillaceae (93%)	Rhodospirillaceae (93%)	Rhodospirillaceae (65%)	Rhodospirillaceae
c58*	Sphingobium	Sphingobium xenophagum (99%)		Sphingomonadales (100%)	Sphingomonadaceae (100%)	Sphingomonadales (70%)	Sphingobium

* Bins c53 and c40x have been merged to a newly defined bin c58

Supplementary Table 5. Average nucleotide identity (ANI) analysis of the 22 MAGs.

MAG	Most similar genome	ANI	Assembly	Genomes tested
c0	*Haliea salexigens* DSM 19537	77.38%	GCA_000423125.1	Each Alteromonadaceae genus
c3	*Erythrobacter citreus* LAMA 915	80.77%	GCA_001235865.1	Each Sphingomonadales genus
c4a	*Desulfotomaculum gibsoniae* DSM 7213	78.77%	GCA_000233715.3	All *Desulfotomaculum* and *Pelotomaculum*
c4b	*Desulfotomaculum gibsoniae* DSM 7213	82.44%	GCA_000233715.3	All *Desulfotomaculum* and *Pelotomaculum*
c7a	--- *No genome match* ---			15 top NCBI BLASTN hits of 16S rRNA gene(s)
c8a	*Desulfotomaculum alcoholivorax* DSM 16058	78.00%	GCA_000430885.1	All *Desulfotomaculum* and *Pelotomaculum*
c9	*Hoeflea* sp. BAL378	83.47%	GCA_000759435.1	All *Hoeflea*
c12	*Desulfococcus multivorans* DSM 2059	76.95%	GCA_000422185.1	15 top NCBI BLASTN hits of 16S rRNA gene(s)
c16a	*Desulfocapsa sulfexigens* DSM 10523	76.30%	GCA_000341395.1	15 top NCBI BLASTN hits of 16S rRNA gene(s)
c19	Desulfitibacter alkalitolerans DSM 16504	77.50%	GCA_000620305.1	All *Desulfitibacter*
c20a	--- *No genome match* ---			15 top NCBI BLASTN hits of 16S rRNA gene(s)
c22	*Hyphomonas oceanitis* SCH89	83.31%	GCA_000685295.1	All *Hyphomonas*
c23	*Desulfotomaculum gibsoniae* DSM 7213	80.88%	GCA_000233715.3	All Peptococcaceae
c25	--- *No genome match* ---			Each Clostridiales family, each Clostridiaceae genus, all *Alkaliphilus*
c29	*Hyphomonas* sp. BH-BN04-4	83.06%	GCA_000682695.1	All Hyphomonadaceae
c32	*Ignavibacterium album* strain JCM 16511	70.14%	GCA_000258405.1	15 top NCBI BLASTN hits of 16S rRNA gene(s)
c36	Candidatus *Filomicrobium marinum* Y	75.86%	GCA_000981545.1	Each Rhizobiales genus
c41	*Roseovarius* sp. 217	98.07%	GCA_000152845.1	All *Roseovarius*
c42	*Porphyrobacter cryptus* DSM 12079	77.91%	GCA_000422985.1	Each Sphingomonadales genus
c52	*Tenacibaculum maritimum* NBRC 15946	76.67%	GCA_000509405.1	15 top NCBI BLASTN hits of 16S rRNA gene(s)
c54	*Crocinitomix catalasitica* ATCC 23190	73.33%	GCA_000621625.1	15 top NCBI BLASTN hits of 16S rRNA gene(s)
c57	*Caenispirillum salinarum* AK4	77.15%	GCA_000315795.1	15 top NCBI BLASTN hits of 16S rRNA gene(s)

Supplementary Table 6. Number of proteins detected (compared to the number of protein-coding genes) for each bin, and the sum of normalized spectral counts for each bin, and their relative proportion among the different bins. Blue shading highlights MAGs present in Fig. 6A and grey shading highlights other MAGs.

Bin	Number of protein-coding genes	Number of proteins detected	Proportion of proteins detected in genomes	Sum of normalized spectral counts	Proportion of normalized spectral counts
c16a	6052	308	5.09%	431273	44.62%
c22	4162	120	2.88%	140944	14.58%
c23	6261	103	1.65%	113801	11.77%
c12	5509	56	1.02%	33778	3.49%
c37	6377	46	0.72%	27191	2.81%
c20b	57	1	1.75%	26417	2.73%
c4a	3558	43	1.21%	24628	2.55%
c57	3954	41	1.04%	23564	2.44%
c8a	4099	42	1.02%	20088	2.08%
c49	6342	21	0.33%	16076	1.66%
c27	2362	7	0.30%	15738	1.63%
c20a	4731	31	0.66%	11061	1.14%
c35	3332	7	0.21%	9124	0.94%
c41	5037	22	0.44%	8849	0.92%
c4c	3138	16	0.51%	8648	0.89%
c21	1215	7	0.58%	6376	0.66%
c7a	4144	10	0.24%	5436	0.56%
c29	3724	18	0.48%	4549	0.47%
c54	2577	6	0.23%	4286	0.44%
c36	3728	7	0.19%	4162	0.43%
c52	3002	6	0.20%	3707	0.38%
c19	3847	13	0.34%	2939	0.30%
c4b	3755	12	0.32%	2862	0.30%
c2	1724	9	0.52%	2607	0.27%
c25	5255	10	0.19%	2505	0.26%
c32	2971	7	0.24%	2455	0.25%
c11	3687	7	0.19%	2184	0.23%
c58	4535	4	0.09%	1898	0.20%
c46	2181	6	0.28%	1628	0.17%
c0	3368	9	0.27%	1526	0.16%
c5	2886	6	0.21%	1524	0.16%
c40	2976	3	0.10%	1109	0.11%
c38	1711	3	0.18%	981	0.10%

Bin	Number of protein-coding genes	Number of proteins detected	Proportion of proteins detected	Sum of normalized spectral counts	Proportion of normalized spectral counts
c9	4057	4	0.10%	750	0.08%
c13	1920	4	0.21%	541	0.06%
c3	2592	3	0.12%	427	0.04%
c43	2334	3	0.13%	359	0.04%
c42	2289	3	0.13%	213	0.02%
c10	627	1	0.16%	75	0.01%
c34	379	1	0.26%	75	0.01%
c50	1637	1	0.06%	75	0.01%
c55	900	1	0.11%	75	0.01%
c16b	151	1	0.66%	28	0.00%
c1	679	0	0.00%	0	0.00%
c6	24	0	0.00%	0	0.00%
c14	243	0	0.00%	0	0.00%
c15	149	0	0.00%	0	0.00%
c17	7	0	0.00%	0	0.00%
c18	332	0	0.00%	0	0.00%
c24	418	0	0.00%	0	0.00%
c26	370	0	0.00%	0	0.00%
c28	1	0	0.00%	0	0.00%
c30	286	0	0.00%	0	0.00%
c31	805	0	0.00%	0	0.00%
c33	43	0	0.00%	0	0.00%
c39	198	0	0.00%	0	0.00%
c44	70	0	0.00%	0	0.00%
c45	58	0	0.00%	0	0.00%
c47	329	0	0.00%	0	0.00%
c48	62	0	0.00%	0	0.00%
c51	1348	0	0.00%	0	0.00%
c56	49	0	0.00%	0	0.00%
c7b	199	0	0.00%	0	0.00%
c8b	33	0	0.00%	0	0.00%
c8c	10	0	0.00%	0	0.00%

Supplementary Table 7. List of proteins involved in processes described in Fig. 5. In the second column, P stands for proteome, while G for genome.

Reaction no.	Protein coding gene ID	In proteome (P) or genome (G) only ?	Subcellulare localization	Gene product	COG number	KO number	EC number
1	BRHa_100687050	P	Cytoplasmic membrane	Ni,Fe-hydrogenase I small subunit	COG1740	K06282	EC:1.12.99.6
1	BRHa_100687051	P	Cytoplasmic membrane	Ni,Fe-hydrogenase I large subunit	COG0374	K06281	EC:1.12.99.6
2	BRHa_100267644	P	Unknown	Heterodisulfide reductase, subunit A and related polyferredoxins	COG1148	K03388	EC:1.8.98.1
2	BRHa_100267645	P	Cytoplasm	Heterodisulfide reductase, subunit A and related polyferredoxins	COG1148	K03388	EC:1.8.98.1
2	BRHa_100267646	P	Cytoplasmic membrane	Heterodisulfide reductase, subunit C	COG1150	-	-
3	BRHa_1004263109	G	Cytoplasmic membrane	Polysulphide reductase	COG5557	K00185	EC:1.2.7.-
3	BRHa_1004263110	G	Unknown	Fe-S-cluster-containing hydrogenase components 1	COG0437	-	-
3	BRHa_1004263112	P	Cytoplasm	Fe-S oxidoreductase	COG0247	-	-
3	BRHa_1004263113	P	Cytoplasmic membrane	Nitrate reductase gamma subunit	COG2181	-	-
4	BRHa_100252619	P	Cytoplasm	ATP sulfurylase (sulfate adenylyltransferase)	COG2046	K00958	EC:2.7.7.4
5	BRHa_100267642	P	Unknown	Adenosine-5'-phosphosulfate reductase beta subunit;	COG4231	K00395	EC:1.8.99.2
5	BRHa_100267643	P	Cytoplasm	Succinate dehydrogenase/fumarate reductase, flavoprotein subunit	COG1053	K00394	EC:1.8.99.2
6	BRHa_100622935	P	Cytoplasm	Dissimilatory sulfite reductase (desulfoviridin), alpha and beta subunits	COG2221	K11180	EC:1.8.99.3
6	BRHa_100622936	P	Cytoplasm	Dissimilatory sulfite reductase (desulfoviridin), alpha and beta subunits	COG2221	K11181	EC:1.8.99.3
6	BRHa_100622937	G	Unknown	Dissimilatory sulfite reductase D (DsrD)	-	-	-
7	BRHa_100622972	P	Unknown	F0F1-type ATP synthase, subunit b	COG0711	K02109	EC:3.6.3.14
7	BRHa_100622973	P	Unknown	F0F1-type ATP synthase, subunit b	COG0711	K02109	EC:3.6.3.14
7	BRHa_100622974	P	Cytoplasm	F0F1-type ATP synthase, delta subunit (mitochondrial oligomycin sensitivity protein)	COG0712	K02113	EC:3.6.3.14
7	BRHa_100622975	P	Cytoplasm	F0F1-type ATP synthase, alpha subunit	COG0056	K02111	EC:3.6.3.14
7	BRHa_100622976	G	Cytoplasm	F0F1-type ATP synthase, gamma subunit	COG0224	K02115	EC:3.6.3.14
7	BRHa_100622977	P	Cytoplasm	F0F1-type ATP synthase, beta subunit	COG0055	K02112	EC:3.6.3.14
7	BRHa_100622978	P	Unknown	F0F1-type ATP synthase, epsilon subunit (mitochondrial delta subunit)	COG0355	K02114	EC:3.6.3.14
7	BRHa_100622955	G	Cytoplasmic membrane	F0F1-type ATP synthase, subunit c/Archaeal/vacuolar-type H+-ATPase, subunit K	COG0636	K02110	EC:3.6.3.14
7	BRHa_100622954	G	Cytoplasmic membrane	F0F1-type ATP synthase, subunit a	COG0356	K02108	EC:3.6.3.14
8	BRHa_1001515120	P	Cytoplasm	Coenzyme F420-reducing hydrogenase, gamma subunit	COG1941	K14128	EC:1.12.99.-
8	BRHa_1001515121	P	Cytoplasm	Coenzyme F420-reducing hydrogenase, alpha subunit	COG3259	K14126	EC:1.12.99.-
9	BRHa_1007437112	P	Cytoplasm	2-polyprenylphenol hydroxylase and related flavodoxin oxidoreductases	COG0543	K00528	EC:1.18.1.2
10	BRHa_100617011	P	Cytoplasm	NADPH:quinone reductase and related Zn-dependent oxidoreductases	COG0604	K00344	EC:1.6.5.5
11	BRHa_10068707	P	Cytoplasmic membrane	Predicted NADH:ubiquinone oxidoreductase, subunit RnfB - COG2878	COG2878	K03616	-
11	BRHa_10068708	P	Cytoplasmic membrane	Predicted NADH:ubiquinone oxidoreductase, subunit RnfA - COG4657	COG4657	K03617	-
11	BRHa_10068709	G	Cytoplasmic membrane	Predicted NADH:ubiquinone oxidoreductase, subunit RnfE - COG4660	COG4660	K03613	-
11	BRHa_100687010	G	Cytoplasm	Predicted NADH:ubiquinone oxidoreductase, subunit RnfG - COG4659	COG4659	K03612	-
11	BRHa_100687011	G	Cytoplasmic membrane	Predicted NADH:ubiquinone oxidoreductase, subunit RnfD - COG4658	COG4658	K03614	-
11	BRHa_100687012	P	Cytoplasm	Predicted NADH:ubiquinone oxidoreductase, subunit RnfC - COG4656	COG4656	K03615	-
12	BRHa_1007437101	P	Cytoplasm	Carbonic anhydrases/acetyltransferases, isoleucine patch superfamily	COG0663	-	-
13	BRHa_100354542	G	Cytoplasm	Uncharacterized anaerobic dehydrogenase	COG3383	K00123	EC:1.2.1.2
13	BRHa_100354543	P	Cytoplasm	Uncharacterized anaerobic dehydrogenase	COG3383	K00123	EC:1.2.1.2
14	BRHa_100354519	P	Cytoplasm	Formyltetrahydrofolate synthetase	COG2759	K01938	EC:6.3.4.3
15-16	BRHa_100354520	P	Cytoplasm	5,10-methylene-tetrahydrofolate dehydrogenase/Methenyl tetrahydrofolate cyclohydrolase	COG0190	K01491	EC:1.5.1.5
17	BRHa_10037249	P	Cytoplasm	5,10-methylenetetrahydrofolate reductase	COG0685	K00297	EC:1.5.1.20
18	BRHa_100354562	P	Cytoplasm	Pterin binding enzyme	COG1410	-	-
19	BRHa_100354559	P	Cytoplasm	6Fe-6S prismane cluster-containing protein	COG1151	K00198	EC:1.2.99.2
19	BRHa_100354565	P	Cytoplasm	6Fe-6S prismane cluster-containing protein	COG1151	K00190	EC:1.2.7.4
20	BRHa_100354566	P	Cytoplasm	CO dehydrogenase/acetyl-CoA synthase delta subunit (corrinoid Fe-S protein)	COG2069	K00194	EC:2.1.1.245
20	BRHa_100354563	P	Unknown	CO dehydrogenase/acetyl-CoA synthase gamma subunit (corrinoid Fe-S protein)	COG1456	K00197	EC:2.1.1.245
20	BRHa_100354564	P	Cytoplasm	CO dehydrogenase/acetyl-CoA synthase beta subunit	COG1614	K14138	EC:1.2.7.4
21	BRHa_1001804275	P	Unknown	Pyruvate:ferredoxin oxidoreductase and related 2-oxoacid:ferredoxin oxidoreductases, alpha subunit	COG0674	K03737	EC:1.2.7.-
21	BRHa_1000308122	P	Unknown	Pyruvate:ferredoxin oxidoreductase and related 2-oxoacid:ferredoxin oxidoreductases, alpha subunit	COG0674	K00169	EC:1.2.7.1
22	BRHa_1004263141	P	Cytoplasm	Pyruvate carboxylase	COG1038	K01958	EC:6.4.1.1
22	BRHa_100115516	P	Periplasm	Pyruvate/oxaloacetate carboxyltransferase	COG5016	K01960	EC:6.4.1.1
22	BRHa_100227747	P	Cytoplasm	Pyruvate/oxaloacetate carboxyltransferase	COG5016	K01960	EC:6.4.1.1
23	BRHa_100332486	P	Cytoplasm	Citrate synthase	COG0372	K01647	EC:2.3.3.1
24	BRHa_100042063	G	Cytoplasm	Aconitase B	COG1049	K01682	EC:4.2.1.3
24	BRHa_100332494	G	Cytoplasm	Aconitase B	COG1049	K01682	EC:4.2.1.3
24	BRHa_100598884	G	Cytoplasm	Aconitase A	COG1048	K01681	EC:4.2.1.-
25	BRHa_100255622	G	Cytoplasm	Isocitrate dehydrogenases	COG0538	K00031	EC:1.1.1.42
25	BRHa_100042058	G	Cytoplasm	Isocitrate dehydrogenases	COG0538	K00031	EC:1.1.1.42
26	BRHa_100598858	P	Cytoplasm	Pyruvate:ferredoxin oxidoreductase and related 2-oxoacid:ferredoxin oxidoreductases, alpha subunit	COG0674	K00174	EC:1.2.7.3
26	BRHa_100598857	G	Cytoplasm	Pyruvate:ferredoxin oxidoreductase and related 2-oxoacid:ferredoxin oxidoreductases, beta subunit	COG1013	K00175	EC:1.2.7.3
26	BRHa_100598856	G	Unknown	Pyruvate:ferredoxin oxidoreductase and related 2-oxoacid:ferredoxin oxidoreductases, gamma subunit	COG1014	K00177	EC:1.2.7.3
26	BRHa_100598859	G	Unknown	-	COG1152	K00176	EC:1.2.7.3
27	BRHa_100227751	P	Cytoplasm	Succinyl-CoA synthetase, alpha subunit	COG0074	K01902	EC:6.2.1.5
27	BRHa_100227752	P	Cytoplasm	Succinyl-CoA synthetase, beta subunit	COG0045	K01903	EC:6.2.1.5
28	BRHa_100705860	G	Cytoplasmic membrane	Succinate dehydrogenase/fumarate reductase, flavoprotein subunit	COG1053	K00239	EC:1.3.99.1
28	BRHa_100705861	P	Cytoplasmic membrane	Succinate dehydrogenase/fumarate reductase, Fe-S protein subunit	COG0479	K00240	EC:1.3.99.1
28	BRHa_100705859	G	Cytoplasmic membrane	-	-	K00241	-
29	BRHa_100029439	G	Cytoplasm	Tartrate dehydratase alpha subunit/Fumarate hydratase class I, N-terminal domain	COG1951	K01676	EC:4.2.1.2
29	BRHa_100029420	G	Cytoplasm	Fumarase	COG0114	K01679	EC:4.2.1.2
30	BRHa_1001804178	P	Unknown	Malate/lactate dehydrogenases	COG0039	K00024	EC:1.1.1.37
30	BRHa_100372494	P	Unknown	Malate/lactate dehydrogenases	COG0039	K00024	EC:1.1.1.37
31	BRHa_100267631	P	Cytoplasm	Phosphoenolpyruvate carboxykinase (GTP)	COG1274	K01596	EC:4.1.1.32
32	BRHa_1000308196	P	Cytoplasm	Enolase	COG0148	K01689	EC:4.2.1.11
33	BRHa_100151541	G	Cytoplasm	Predicted phosphoglycerate mutase, AP superfamily - COG3635	COG3635	K15635	EC:5.4.2.1
33	BRHa_100204380	G	Cytoplasm	Phosphoglyceromutase	COG0696	K01834	EC:5.4.2.1
33	BRHa_100348113	G	Cytoplasm	Fructose-2,6-bisphosphatase	COG0406	K01834	EC:5.4.2.1
34	BRHa_1003749104	G	Cytoplasm	3-phosphoglycerate kinase	COG0126	K00927	EC:2.7.2.3
35	BRHa_100622964	P	Cytoplasm	Glyceraldehyde-3-phosphate dehydrogenase/erythrose-4-phosphate dehydrogenase	COG0057	K00134	EC:1.2.1.12
35	BRHa_1003749106	P	Cytoplasm	Glyceraldehyde-3-phosphate dehydrogenase/erythrose-4-phosphate dehydrogenase	COG0057	K00134	EC:1.2.1.12
36	BRHa_1003749103	G	Cytoplasm	Triosephosphate isomerase	COG0149	K01803	EC:5.3.1.1
37	BRHa_1001515284	P	Cytoplasm	Fructose/tagatose bisphosphate aldolase	COG0191	K01624	EC:4.1.2.13
38	BRHa_1001804297	G	Cytoplasm	Archaeal fructose 1,6-bisphosphatase	COG1980	K01622	EC:3.1.3.11

Supplementary Table 7. (continued)

Reaction no.	Protein coding gene ID	In proteome (P) or genome (G) only ?	Subcellulare localization	Gene product	COG number	KO number	EC number
39	BRHa_100227759	P	Cytoplasm	Acyl-coenzyme A synthetases/AMP-(fatty) acid ligases	COG0365	K01895	EC:6.2.1.1
39	BRHa_100622969	P	Cytoplasm	Acyl-coenzyme A synthetases/AMP-(fatty) acid ligases	COG0365	K01895	EC:6.2.1.1
40	BRHa_100227744	P	Cytoplasm	Methylmalonyl-CoA mutase, N-terminal domain/subunit	COG1884	K01847	EC:5.4.99.2
41	BRHa_1003324154	P	Cytoplasm	Acetyl-CoA carboxylase, carboxyltransferase component (subunits alpha and beta)	COG4799	K01966	EC:6.4.1.3
41	BRHa_100227750	P	Cytoplasm	Acetyl-CoA carboxylase, carboxyltransferase component (subunits alpha and beta)	COG4799	K01966	EC:6.4.1.3
41	BRHa_1003324155	P	Cytoplasm	Acetyl/propionyl-CoA carboxylase, alpha subunit	COG4770	K01965	EC:6.4.1.3
42	BRHa_100227753	P	Cytoplasm	Acyl-coenzyme A synthetases/AMP-(fatty) acid ligases	COG0365	K01908	EC:6.2.1.17
43	BRHa_1004263142	P	Cytoplasm	Pyruvate carboxylase	COG1038	K01961	EC:6.3.4.14
43	BRHa_100082530	G	Cytoplasm	Acetyl-CoA carboxylase alpha subunit	COG0825	K01962	EC:6.4.1.2
44	BRHa_100227733	P	Cytoplasm	Malonyl-CoA decarboxylase (MCD)	-	K01578	EC:4.1.1.9
45	BRHa_100252659	P	Cytoplasm	(acyl-carrier-protein) S-malonyltransferase	COG0331	K00645	EC:2.3.1.39
45	BRHa_100227720	G	Cytoplasm	3-oxoacyl-[acyl-carrier-protein] synthase III	COG0332	K00648	EC:2.3.1.180
45	BRHa_10011556	G	Cytoplasm	3-oxoacyl-[acyl-carrier-protein] synthase III	COG0332	K00648	EC:2.3.1.180
45	BRHa_1003324146	G	Cytoplasm	3-oxoacyl-[acyl-carrier-protein] synthase III	COG0332	K00648	EC:2.3.1.180
46	BRHa_1000308102	P	Cytoplasm	Acetyl-CoA acetyltransferase	COG0183	K00626	EC:2.3.1.9
46	BRHa_1003749180	P	Cytoplasm	Acetyl-CoA acetyltransferase	COG0183	K00626	EC:2.3.1.9
47	BRHa_1001804165	P	Cytoplasmic membrane	Long-chain acyl-CoA synthetases (AMP-forming)	COG1022	K01897	EC:6.2.1.3
47	BRHa_100372481	P	Cytoplasmic membrane	Long-chain acyl-CoA synthetases (AMP-forming)	COG1022	K01897	EC:6.2.1.3
48	BRHa_100613146	P	Cytoplasm	Acetyl-CoA acetyltransferase	COG0183	K00632	EC:2.3.1.16
49	BRHa_100598844	P	Outer membrane	Long-chain fatty acid transport protein	COG2067	K06076	-
50	BRHa_100598838	P	Cytoplasm	Acyl-CoA dehydrogenases	COG1960	K00248	EC:1.3.99.2
50	BRHa_100598839	P	Cytoplasmic membrane	Fe-S oxidoreductase	COG0247	-	-
51	BRHa_100227716	P	Cytoplasm	Dehydrogenases with different specificities (related to short-chain alcohol dehydrogenases)	COG1028	K00059	EC:1.1.1.100
52	BRHa_100111212	P	Cytoplasm	Ketol-acid reductoisomerase	COG0059	K00053	EC:1.1.1.86
53	BRHa_100151542	P	Cytoplasm	Homoserine dehydrogenase	COG0460	K00003	EC:1.1.1.3
54	BRHa_100297220	P	Cytoplasm	Isocitrate/isopropylmalate dehydrogenase	COG0473	K00052	EC:1.1.1.85
55	BRHa_1001515241	P	Cytoplasm	Phosphoglycerate dehydrogenase and related dehydrogenases	COG0111	K00058	EC:1.1.1.95
55	BRHa_100722337	P	Cytoplasm	Phosphoglycerate dehydrogenase and related dehydrogenases	COG0111	K00058	EC:1.1.1.95
56	BRHa_100725848	P	Cytoplasm	Dihydrodipicolinate reductase	COG0289	K00215	EC:1.3.1.26
57	BRHa_1007437113	P	Cytoplasm	NADPH-dependent glutamate synthase beta chain and related oxidoreductases	COG0493	K00266	EC:1.4.1.13
58	BRHa_100725854	P	Cytoplasm	Ornithine carbamoyltransferase	COG0078	K00611	EC:2.1.3.3
59	BRHa_1004263104	P	Cytoplasm	5-enolpyruvylshikimate-3-phosphate synthase	COG0128	K00800	EC:2.5.1.19
60	BRHa_100725855	P	Cytoplasm	Ornithine/acetylornithine aminotransferase	COG4992	K00818	EC:2.6.1.11
61	BRHa_100541474	P	Cytoplasm	Glucosamine 6-phosphate synthetase	COG0449	K00820	EC:2.6.1.16
62	BRHa_100705853	P	Cytoplasm	Phosphoserine aminotransferase	COG1932	K00831	EC:2.6.1.52
63	BRHa_1007437171	P	Cytoplasm	Aspartate/tyrosine/aromatic aminotransferase	COG1448	K00832	EC:2.6.1.57
64	BRHa_1007437174	P	Cytoplasm	Aspartokinases	COG0527	K00928	EC:2.7.2.4
65	BRHa_100297219	P	Cytoplasm	Phosphoribosylpyrophosphate synthetase	COG0462	K00948	EC:2.7.6.1
66	BRHa_1007437130	P	Cytoplasm	Thiamine pyrophosphate-requiring enzymes	COG0028	K01652	EC:2.2.1.6
67	BRHa_100725849	P	Cytoplasm	Dihydrodipicolinate synthase/N-acetylneuraminate lyase	COG0329	K01714	EC:4.3.3.7
68	BRHa_100722334	P	Cytoplasm	Peptidyl-prolyl cis-trans isomerase (rotamase) - cyclophilin family	COG0652	K01802	EC:5.2.1.8
69	BRHa_1006229124	P	Cytoplasm	Glutamine synthetase	COG0174	K01915	EC:6.3.1.2
70	BRHa_100725853	P	Cytoplasm	Argininosuccinate synthase	COG0137	K01940	EC:6.3.4.5
71	BRHa_100705865	P	Cytoplasm	Aspartate/tyrosine/aromatic aminotransferase	COG0436	K10206	EC:2.6.1.83
72	BRHa_100372414	P	Cytoplasm	NADPH-dependent glutamate synthase beta chain and related oxidoreductases	COG0493	-	-
73	BRHa_100354510	P	Cytoplasm	Aspartate-semialdehyde dehydrogenase	COG0136	K00133	EC:1.2.1.11
74	BRHa_100255635	P	Cytoplasm	Saccharopine dehydrogenase and related proteins	COG1748	K00290	EC:1.5.1.7
75	BRHa_100725845	P	Cytoplasm	Transaldolase	COG0176	K00616	EC:2.2.1.2
76	BRHa_100722359	P	Cytoplasm	Threonine synthase	COG0498	K01733	EC:4.2.3.1
77	BRHa_100687040	P	Cytoplasm	Predicted ornithine cyclodeaminase, mu-crystallin homolog - COG2423	COG2423	K01750	EC:4.3.1.12
78	BRHa_100426372	P	Cytoplasm	O-acetylhomoserine sulfhydrylase	COG2873	K01740	EC:2.5.1.49
79	BRHa_100146147	P	Extracellular	Flagellin and related hook-associated proteins	COG1344	K02406	-
79	BRHa_100146156	P	Extracellular	Flagellin and related hook-associated proteins	COG1344	K02406	-
80	BRHa_10025264	P	Extracellular	-	COG4968	-	-
81	BRHa_100297236	P	Unknown	Tfp pilus assembly protein, tip-associated adhesin PilY1	COG3419	-	-
81	BRHa_100252616	P	Unknown	-	COG3419	K02674	-
82	BRHa_1003749111	P	Cytoplasm	Tfp pilus assembly protein, ATPase PilM	COG4972	K02662	-
83	BRHa_1003749115	P	Outer membrane	-	COG4796	K02666	-
84	BRHa_100722394	P	Cytoplasmic membrane	Sulfate permease and related transporters (MFS superfamily)	COG0659	-	-
85	BRHa_100676489	P	Periplasm	ABC-type branched-chain amino acid transport systems, periplasmic component	COG0683	K01999	-
85	BRHa_100332442	P	Periplasm	ABC-type branched-chain amino acid transport systems, periplasmic component	COG0683	K01999	-
85	BRHa_100705819	P	Periplasm	ABC-type branched-chain amino acid transport systems, periplasmic component	COG0683	K01999	-
85	BRHa_100657099	P	Periplasm	ABC-type branched-chain amino acid transport systems, periplasmic component	COG0683	K01999	-
85	BRHa_100594516	P	Periplasm	ABC-type branched-chain amino acid transport systems, periplasmic component	COG0683	K01999	-
85	BRHa_100426345	P	Unknown	ABC-type branched-chain amino acid transport systems, periplasmic component	COG0683	K01999	-
86	BRHa_100297226	P	Periplasm	ABC-type amino acid transport/signal transduction systems, periplasmic component/domain	COG0834	K09969	-
87	BRHa_100030891	P	Periplasm	ABC-type amino acid transport/signal transduction systems, periplasmic component/domain	COG0834	K02030	-
88	BRHa_1003724106	P	Cytoplasmic membrane	ABC-type polar amino acid transport system, ATPase component	COG1126	K02028	EC:3.6.3.21
92	BRHa_100692244	P	Cytoplasm	Inorganic pyrophosphatase/exopolyphosphatase	COG1227	K01507	EC:3.6.1.1
93	BRHa_1006081109	P	Cytoplasm	Phosphate uptake regulator	COG0704	K02039	-
93	BRHa_10074317	P	Cytoplasm	Phosphate uptake regulator	COG0704	K02039	-
90	BRHa_1006081105	P	Cytoplasmic membrane	ABC-type phosphate transport system, ATPase component	COG1117	K02036	EC:3.6.3.27
90	BRHa_100743111	P	Cytoplasmic membrane	ABC-type phosphate transport system, ATPase component	COG1117	K02036	EC:3.6.3.27
91	BRHa_100743110	P	Cytoplasmic membrane	ABC-type phosphate transport system, periplasmic component	COG0226	K02040	-
91	BRHa_1006081106	P	Cytoplasmic membrane	ABC-type phosphate transport system, periplasmic component	COG0226	K02040	-
89	BRHa_100393024	P	Periplasm	ABC-type phosphate/phosphonate transport system, periplasmic component	COG3221	K02044	-
94	BRHa_100151530	P	Periplasm	ABC-type Fe3+ transport system, periplasmic component	COG1840	K02012	-
95	BRHa_1003749212	P	Cytoplasm	Ferritin-like protein	COG1528	K02217	EC:1.16.3.1
96	BRHa_1003724142	P	Unknown	ABC-type sugar transport system, periplasmic component	COG1653	K02027	-
96	BRHa_1001804226	P	Unknown	ABC-type_sugar_transport_system,_periplasmic_component	COG1653	K02027	-

SUPPLEMENTARY METHODS

Instrumented H_2 injection borehole

A 25 m long borehole was drilled from the gallery floor (borehole BRC-3). A hydraulic neoprene packer was installed at the bottom of the borehole, in order to create a 2.74 m long chamber isolated from oxic gallery atmosphere, where porewater constantly produced by the borehole (at a rate of 20 mL/day) accumulated. Multiple polyamide lines were placed for connecting this chamber to surface equipment, allowing water recirculation and sampling. To avoid lines clogging with particles, a PVC screen was also installed in the chamber. An artist's rendering of the borehole equipment in presented in Fig. 2.

The surface equipment, through which borehole water was recirculated, consists of PEEK lines connected in a circulation loop to a plexiglas sediment trap (originally designed as sampling cylinders), a peristaltic pump (with Pharmed BPT tubing), a flow-meter, a dissolved oxygen probe, a gas permeable membrane connected to a 500 mL reservoir filled with 100% H_2 and two needle valves, the first one placed right after borehole and second one right before the borehole, in the direction of water flow (Supplementary Fig. 7). In order to protect this experiment from oxygen contamination when borehole water was recirculated, a plexiglas cabinet was installed and was regularly flushed with argon. In addition to the circulation loop, another line was dedicated to borehole pressure monitoring, releasing water when pressure was above 0.5 bars (relative pressure). Pure H_2 was later directly and non-continuously injected into the borehole chamber, thus creating a gas phase. More details concerning experiment set-up of recirculation and non-recirculation modes can be found in Supplementary Fig. 7 and in paragraphs describing sampling procedures (below).

It was not possible to install this experimental setting under truly sterile conditions. However, great care was taken to limit contamination. These steps include, depending on the material, autoclaving, ethanol flaming, rinsing with 70 % ethanol, 1 M HCl (overnight), or 7 % bleach, before rinsing with sterile water.

During recirculation mode, samples were recovered by connecting a sterile and anoxic 1 L bottle to needle valve 1 when the pump was shut down. This sample, which consists of several hundred mL of borehole water, was mainly used for DNA extraction, but also served for some chemical analyses. For some other analysis, borehole water was directly sampled from needle valves. More information about this particular type of sampling can be found in the next section. Borehole overpressure was used as a driving force for water sampling. But,

in order to keep borehole pressure constant, sterile and anoxic artificial porewater (APW), amended with HCO_3^- right before use, whose chemical composition mimics the one of the natural porewater composition, was injected into needle valve 2, from a sealed bottle connected to a sterile argon flux whose pressure was 0.5 relative bar. In this way, pressure and water volume in borehole stayed constant during sampling. In order to avoid sampling this artificial porewater, all connections between needle valves 1 and 2 in surface equipment were closed, forcing the water flow to go from artificial porewater bottle, to the interval and to the sampling bottle. Tracer tests were carried out to figure out how much borehole water can be sampled before injected artificial porewater is sampled.

During non-recirculation mode, in order not to sample the water contained in the lines dead volume, pump was either started for 20 min at 5 mL/min, or the first 100 mL of water were discarded. Pressure regulating line was kept closed. The higher borehole pressure caused by the injection of H_2 allowed for a larger sampling volume even before any artificial porewater injection. However, to sample the last hundred milliliters, when borehole pressure got closer to atmospheric pressure, artificial porewater was injected, as described earlier, to increase borehole pressure. This injection continued until after the sampling was done, in order to replace all the sampled water. After this injection, 1 to 3 L of sterile H_2 was injected to borehole through needle valve 2.

We used a third mode, which consisted of a transition between the two modes described above: borehole water was recirculated but H_2 was non-continuously injected into the borehole. In term of sampling and monitoring, this mode is similar to the recirculation mode.

APW composition is given in Supplementary Table 1. The composition of APW I, used until day 324, was based on porewater modeling for another borehole located nearby in the Underground Rock Laboratory (URL) [1]. Starting on day 324, it was decided to use an APW matching the ionic composition of natural porewater from BRC-3 better. The APW II composition was based on ion chromatograph measurements from BRC-3 porewater that was sampled before the experiment started.

Chemical sampling and assays

To measure organic acids, a 1.5 to 6 mL aliquot was withdrawn from the 1 L sampling bottle, filtered with a 0.2 µm pore size filter and stored at -20 ˚C until analysis. Before analysis, a volume of 4.5 mL of APW II was added (if needed) to complete the sample volume to 6 mL. 1 M $BaCl_2$ solution (150 µL) was added, in order to precipitate SO_4^{2-}. After a 10 min

incubation, samples were filtered through a 0.2 µm filter and passed through OnGuard Ag cartridges 1cc and OnGuard H cartridges 1cc (Dionex, Sunnyvale, USA) according to manufacturer's protocol, in order to remove chloride ions. 1M NaOH solution (3 µL) was then added to 1.5 mL of treated samples. Finally, samples were measured by ion chromatography (DX-3000, Dionex, Sunnyvale, USA), using an IonPac AS11-HC column and 0.5 mM KOH solution for the elution.

For each sampling session, S(-II) was measured by averaging values obtained for several sub-samples from the 1 L sampling bottle or directly from needle valve 1 or 2, using the method described by Cline [2]. For each sample, about 1.6 mL of borehole water was placed in a 2 mL tube containing 0.4 mL of 50 g/L zinc acetate solution. The exact amount of water sampled was determined by difference in tube weight before and after sampling. Samples were stored at 4 ˚C if they were analyzed within 1 or 2 days, or at -20 ˚C. 80 µL of Cline reagent, which is a 50 % (v/v) HCl solution containing 4 g/L of *N*,*N*-Dimethyl-1,4-phenylenediamine oxalate and 6 g/L of iron(III) chloride hexahydrate, was added to 1 mL of sample. Absorbance was read at 664 nm using a spectrophotometer after an incubation period of 30 min in the dark.

For each sampling session, Fe(II) was measured by averaging values obtained by several sub-samples from the main sampling bottle or directly from needle valve 1 or 2, using the method described by Stookey [3]. For each sample, about 1 mL of borehole water was placed in a 2 mL tube containing 1 mL of 1M HCl solution. The exact amount of water sampled was determined by the difference in tube weight before and after sampling. Samples were stored at 4 ˚C if they were analyzed within 1 or 2 days, or at -20 ˚C. 900 µL of 50 mM HEPES solution (pH 7.0) containing 1 g/L of ferrozine was added to 100 µL of sample. Absorbance was read at 562 nm using a spectrophotometer immediately.

For measuring dissolved gases (H_2 and CO_2), 5 to 10 mL of borehole samples were recovered in a pre-prepared sealed serum bottle. The bottle contained 3.7 mg of mercury(II) chloride, was flushed with N_2, and its internal volume and pressure (null or slightly positive relative to atmospheric pressure) was measured. The samples were directly recovered from needle valves, using a needle. The exact amount of water sampled was determined by the difference in bottle weight before and after sampling. Each sample was stored on its side at 37 ˚C between 1 and 2 days for gas phase equilibration. After this incubation, the gas phase was sampled using a gas-tight syringe and 1 mL was injected in a GC-FID (Varian 450-GC, Agilent, Santa Clara, USA). The H_2 was separated on a 1.5 meter mol sieve 13x 80/100 mesh, and the CH_4 was separated on a 2 meter hayesep Q 80/100 mesh column.

In order to back-calculate the dissolved gas concentration, first the volume of sampled water was determined.

$$volume\ of\ sampled\ water\ [mL] = \frac{bottle\ weigth\ after\ sampling\ [g] - \ bottle\ weight\ before\ sampling\ [g]}{water\ sample\ density\ [g \cdot mL^{-1}]}$$

Then, headspace volume can be calculated.

$$headspace\ volume\ [mL] = \ total\ bottle\ volume\ [mL] - \ volume\ of\ sampled\ water\ [mL]$$

Then, the final pressure of the sampling bottle was calculated.

$$bottle\ pressure\ after\ sampling\ [abs\ bar] = \ bottle\ pressure\ before\ sampling\ [abs\ bar] \times \frac{total\ bottle\ volume\ [mL]}{headspace\ volume\ [mL]}$$

The following calculations were performed for each analyzed gas. The gas partial pressure was calculated.

$$gas\ partial\ pressure\ [Pa] = \ gas\ GC\ measurement\ [\%]\ \times\ bottle\ pressure\ after\ sampling\ [abs\ bar]\ \times\ 10^5$$

Then, the gas concentration in headspace was calculated.

$$gas\ concentration\ in\ headspace\ [M] = \frac{gas\ partial\ pressure\ [Pa]}{8.314\ [J \cdot mol^{-1} \cdot K^{-1}]\ \times\ incubation\ temperature\ [K]} \times \frac{1}{1000}$$

Then, the number of moles of gas in the headspace was calculated.

$$gas\ amount\ in\ headspace\ [mol] = \ gas\ concentration\ in\ headspace\ [M]\ \times\ headspace\ volume\ [mL]/1000$$

Then, Henry's law constant kH,cc, which gives the ratio between gas concentration in water and gas concentration in headspace at equilibrium and for a given temperature, was calculated for each gas and for incubation temperature [4].

$$\begin{aligned} Henry's\ law\ constant\ k_{H,cp}\ [at\ a\ temperature\ T] \\ = k_{H,cp}\ [at\ a\ temperature\ of\ 298.15\ K] \\ \times\ e^{(constant\ C\ of\ van't\ Hoff\ equation\ [K] \times (1/incubation\ temperature\ T\ [K] - 1/298.15\ [K])} \end{aligned}$$

where: $k_{H,cp} = 3.4\ \times\ 10^{-2}$ for CO_2

$k_{H,cp} = 7.8\ \times\ 10^{-4}$ for H_2

$k_{H,cp} = 1.4\ \times\ 10^{-3}$ for CH_4

and where: $constant\ C\ of\ van't\ Hoff\ equation = 2.4 \times 10^3$ for CO_2

$constant\ C\ of\ van't\ Hoff\ equation = 5.0 \times 10^2$ for H_2

$constant\ C\ of\ van't\ Hoff\ equation = 1.6 \times 10^3$ for CH_4

$$Henry's\ law\ constant\ k_{H,cc}\ at\ a\ temperature\ T = k_{H,cp}\ at\ a\ temperature\ T \cdot incubation\ temperature\ T\ [K]/12.2$$

Then, gas concentration in water and after equilibrium can be calculated.

$$gas\ concentration\ in\ water\ after\ equilibrium\ [M] = gas\ concentration\ in\ headspace\ [M] \times Henry's\ law\ constant\ k_{H,cc}\ at\ a\ temperature\ T$$

Then, gas amount in water and after equilibrium can be calculated.

$$gas\ amount\ in\ water\ after\ equilibrium\ [mol] = gas\ concentration\ in\ water\ after\ equilibrium\ [M] \times volume\ of\ sampled\ water\ [mL]/1000$$

Then, the total amount of gas before equilibrium can be calculated.

$$total\ gas\ amount\ before\ equilibrium\ [mol] = gas\ amount\ in\ water\ after\ equilibrium\ [mol] + gas\ amount\ in\ headspace\ [mol]$$

Finally, sample gas concentration can be calculated.

$$gas\ concentration\ in\ sample\ [M] = \frac{total\ gas\ amount\ before\ equilibrium\ [mol]}{volume\ of\ sampled\ water\ [mL]\ /1000}$$

Planktonic cell density

A single sample intended for planktonic cell density analysis was recovered per timepoint from needle valve 1 in a sterile and anoxic serum bottle. The sample was measured within five hours. 1 mL was filtered at 0.2 µm on a black polycarbonate membrane, and then rinsed 3x with PBS buffer (8 g/L of NaCl, 0.2 g/L of KCl, 1.44 g/L of Na_2HPO_4, 0.24 g/L of KH_2PO_4, pH = 7.4). The membrane was then stained with SybrGreen I as described by Lunau *et al.* [5]. For each samples, 15 fields were observed with an epifluorescence Nikon Eclipse E800 microscope at 1,000x magnification and a B-2A filter. In order to account for heterogeneity in the cell distribution on the filter membrane, the reported number of cells represents the average of all 15 fields of view and the standard deviation was calculated from cells counts from 3 groups of 5 images. Planktonic cell density was calculated with this formula:

$$Total\ cell\ concentration\ [\frac{cell}{mL}] = \frac{Mean\ number\ of\ cells \times filter\ area\ [\mu m^2]}{couting\ field\ area\ [\mu m^2] \times sample\ volume\ [mL]}$$

Sampling from other boreholes

Twenty-three samples were collected from 7 other saturated boreholes at the Mont Terri URL (Fig. 1) using three approaches. Most boreholes were equipped with a packer, a large hydraulic stopper that separates borehole water from the oxic atmosphere of the gallery, and long tubing lines that allowed borehole sampling from the gallery. For boreholes with sufficiently high internal pressures, water was sampled directly into a sterile and anoxic bottle after discarding the dead volume of the lines. Where boreholes were not significantly over-pressurized, a sterile and anoxic gas-sampling bag was connected to one of the lines, and the water was recovered over time at rates between 10 and 25 mL per day. Since these bags remained in the gallery for several weeks, oxygen probably diffused through the bag wall, causing slight oxygen contamination in the water sample. A few boreholes lacking a packer (because it was absent or removed prior to sampling) and with a vertical and descending orientation were sampled using a sterile water bottle attached to an aluminum rod sterilized by ethanol and flaming. All water samples were filtered within hours of recovery, using a sterile filtration device and a sterile 0.22 µm polycarbonate membrane. The membrane was frozen at -20°C until DNA extraction.

DNA sampling and extraction

Several hundred milliliters of water samples from the 7 other boreholes were recovered in anoxic and sterile bottles, before filtration using sterile 0.2 µm polycarbonate filters (gentle vacuum filtration), which were immediately placed in a 1.5 mL sterile tube containing 0.4 mL of LifeGuard Soil Preservation Solution (MO BIO Laboratories Inc, Carlsbad, USA), prior to being frozen at -20 ˚C. The DNA of a first set of samples was extracted using a modified version of FastDNA SPIN Kit for Soil (MP Biomedicals, Santa Ana, USA):

- Add the filter to a Lysing Matrix E tube.
- Add 650 µL Sodium Phosphate Buffer to sample in Lysing Matrix E tube.
- Add 80 µL MT Buffer.
- Gently mix the tube by invert it by hand.
- Incubate the tube 5 minutes at 60 °C.
- Homogenize in the FastPrep Instrument for 45 seconds at a speed of 5.5 m/s.
- Centrifuge at 12’000 g for 2 minutes to pellet debris.

- Transfer 400 μl supernatant to a clean 2.0 mL microcentrifuge tube.
- Add 400 μL Sodium Phosphate Buffer to sample in Lysing Matrix E tube.
- Add 50 μL MT Buffer.
- Gently mix the tube by slowly turning it over.
- Incubate the tube 5 minutes at 60 °C.
- Vigorously shake the Lysing Matrix E tube for 45 seconds.
- Centrifuge at 12’000 g for 2 minutes.
- Transfer 500 μL supernatant to the 2.0 ml microcentrifuge tube.
- Add 250 μL PPS (Protein Precipitation Solution).
- Mix by shacking the tube by hand 10 times.
- Centrifuge at 12’000 g for 3 minutes to pellet precipitate.
- Transfer supernatant to a clean 15 mL tube.
- Resuspend Binding Matrix suspension and add 1.0 mL to supernatant in 15 mL tube.
- Place tubes on a rack and invert them by hand for 2 minutes to allow binding DNA.
- Incubate the tube for 3 minutes at room temperature to allow settling of silica matrix.
- Remove and discard 500 μL of supernatant being careful to avoid settled Binding Matrix.
- Resuspend Binding Matrix in the remaining amount of supernatant. Transfer approximately 600 μL of the mixture to a SPIN Filter and centrifuge at 12’000 g for 1 minute. Empty the catch tube.
- Repeat last step again till all the Binding Matrix have been transferred and centrifuged in the SPIN Filter.
- Add 500 μL prepared SEWS-M (with ethanol added) and gently resuspend the pellet using the force of the liquid from the pipet tip.
- Centrifuge at 12’000 g for 1 minute. Empty the catch tube and replace.

- Without any addition of liquid, centrifuge a second time at 12’000 g for 2 minutes to dry the matrix of residual wash solution. Discard the catch tube and replace with a new, clean catch tube.
- Air dry the SPIN Filter for 5 minutes at room temperature.
- Gently resuspend Binding Matrix (above the SPIN Filter) in 100 μL of 10 mM Tris-HCl, pH 7.5.
- Mix, close the tube, and incubated 2 minutes at room temperature.
- Centrifuge at 12’000 g for 1 minute to bring eluted DNA into the clean catch tube. Discard the SPIN Filter.
- Incubate 5 minutes at 60 °C for 5 minutes.
- Separate the DNA in two samples, in 500 μL tubes. One tube can be stored at -80°C and the other can be used or stored at -20°C. If it is used in the 2 days, it can be stored in the fridge.

An extra purification step was carried out subsequently, using the standard protocol of Genomic DNA Clean & Concentrator purification kit (Zymoresearch, Irvine, USA).

Water samples from BRC-3 (where the *in situ* experiment took place) were extracted using a second method that is phenol-chloroform extraction followed by an ethanol precipitation. The reason is that FastDNA SPIN Kit for Soil method recovered poor DNA quality (in term of fragment length) from samples containing S(-II) and black precipitates. It was decided to use a method that doesn’t involve bead-beating because low quality DNA was obtained with that mechanical lysis approach. It starts with the recovery of biomass from filtration membranes. After having filtered and stored the sample in LifeGuard, the membrane was placed in a 60 mL sterile bag containing 0.6 mL of TE buffer pH 7.5-8.0. The bag was closed and biomass was transferred to the TE buffer by rubbing the membrane with one’s fingers on the outside of the bag. The TE buffer containing the filtrate was transferred to a new tube, and combined with the pellet obtained by centrifuging the LifeGuard solution (after having removed the membrane) at 7’000 g for 10 minutes. For DNA extraction, the following procedure was carried out:

- Add lysozyme to 150 mg/L.
- Incubate 2 hours at 37 ˚C.

- Add SDS to 0.1%.
- Freeze/thaw the sample 3 times.
- Treat the lysate with Proteinase K (100 mg/L) for 1 hour at 55 ˚C.
- Add one volume of phenol/chloroform/isoamyl alcohol (pH 7.5-8.0, TE-saturated).
- Mix by inverting rapidly the tubes.
- Centrifuge 1 minute at 14’000 g at room temperature.
- Pipet the aqueous phase to a fresh tube.
- Repeat 4 last steps with the aqueous phase until no protein is visible at the interface of the phases.
- Add to the aqueous phase one volume of chloroform/isoamyl alcohol.
- Mix by inverting rapidly the tubes.
- Centrifuge 1 minute at 14’000 g at room temperature.
- Pipet the aqueous phase to a fresh tube.
- Add 0.1 volume of Na-acteate 3M pH 5.2.
- Add glycogen 1 µL of a solution of 20 g/L of glycogen.
- Add 2 volume of EtOH 100%.
- Mix gently.
- Mix and incubate over night at 4 ˚C or -20 ˚C, or 1 hour at -20 ˚C or -80 ˚C.
- Centrifuge 20 minutes at 16’000 g at (4 ˚C).
- Discard carefully the supernatant.
- Add some 70% EtOH (at -20 ˚C if possible).
- Centrifuge 15 minutes at 16’000 g at 4 ˚C.
- Remove carefully the supernatant and remove all drops around the pellet.
- Let it dry for 10 minutes.
- Resuspend the pellet in TE buffer, pH 7.5-8.0.
- Incubate at 60 ˚C for 15 minutes.

- Freeze the sample at -20 ˚C if needed to be store for more than 2 days (otherwise at 4 ˚C).

16S rRNA gene sequencing

Itag 16S rRNA sequencing was done by the Joint Genome Institute through a community sequencing program project (CSP 1505). Libraries for Illumina MiSeq (San Diego, USA) sequencing (a 2x250 bp reads configuration) were produced by amplifying region V4 of the 16S rRNA gene using primers 515F (5'-GTG CCA GCM GCC GCG GTAA-3') and 806R (5'-GGA CTA CHV GGG TWT CTA AT-3'), with 30 cycles with an annealing temperature of 50°C. Amplicons were then analyzed using the JGI iTagger [6] version 1.1 pipeline (bitbucket.org/berkeleylab/jgi_itagger). However, the two last samples of H_2 enrichment, which were recovered at days 483 and 505, were sequenced at Research and Testing laboratory (Lubbock, USA) and were analyzed by their own bioinformatic method. Paired-end reads were first merged and trimmed back at the last base where the total average is a Phred score greater than 25. USEARCH algorithm [7] (www.drive5.com/usearch/) was then used for dereplicating and clustering the reads longer than 100 bp at a 4% divergence. OTUs are then defined using UPARSE OTU selection algorithm [8] (drive5.com/uparse/) and chimeric sequences were removed using the de novo mode of UCHIME chimera detection software [9] (drive5.com/usearch/manual/uchime_algo.html).

BLAST [10] version 2.2.28 (www.ncbi.nlm.nih.gov/books/NBK1762/) was then used for mapping OTUs of samples recovered at days 483 and 505 days to OTUs from iTagger pipeline, in order to merge all samples in a single biom file. 98.8% of Research and Testing laboratory reads could be mapped to iTaggers OTUs. For the other ones, taxonomic annotation obtained by a USEARCH [7] based method (www.drive5.com/usearch/) using a database of high quality sequences derived from NCBI (www.ncbi.nlm.nih.gov/) was used. An OTU table is presented in Supplementary Data 1.

Metagenomic sequencing and assembly

For samples recovered at days 181, 188, 195, 202, 206, 209, 214, 238, 246 and 250, True seq DNA LT protocol (Illumina, San Diego, USA) was used for library preparation, with 0.5 µg of DNA as a starting material, and with a targeted fragment length of 500 bp. DNA was sequenced using Illumina HiSeq 2500, generating 100 bp paired-end reads at the Lausanne Genomic Technologies Facility. The reads were then quality trimmed with sickle [11] version 1.210 (github.com/najoshi/sickle). To reduce the computational demands of the assembly, the ~2 billion reads were subsequently subsampled to 25 percent with seqtk version 1.0-r32

(github.com/lh3/seqtk). These 10 samples were co-assembled with Ray [12] version 2.3.1 (denovoassembler.sourceforge.net/) using a kmer length of 41 on a Cray XE6 system using 1,024 cores in 2.5 hours.

Samples recovered at days 14, 48, 101, 122, 134 and 233 were sequenced at the Joint Genome Institute (Walnut Creek, USA). For preparing libraries, 100ng of DNA was sheared to 270 bp using the covaris E210 (Covaris, Woburn, USA) and size selected using SPRI beads (Beckman Coulter, Fullerton, USA). The fragments were treated with end-repair, A-tailing, and ligation of Illumina compatible adapters (Integrative DNA Technologies, Coralville, USA) using the KAPA-Illumina library creation kit (KAPA biosystems, Wilmington, USA). qPCR was used to determine the concentration of the libraries and were sequenced on the Illumina Hiseq. For sequencing, the libraries were quantified using KAPA Biosystem's next-generation sequencing library qPCR kit and run on a Roche LightCycler 480 real-time PCR instrument. The quantified libraries were then prepared for sequencing on the Illumina HiSeq sequencing platform utilizing a TruSeq paired-end cluster kit, v3, and Illumina's cBot instrument to generate a clustered flowcell for sequencing. Sequencing of the flowcell was performed on the Illumina HiSeq2000 sequencer using TruSeq SBS sequencing kits, v3, following a 2x150 indexed run recipe. For QC filtering, Raw Illumina metagenomic reads were screened against Illumina artifacts with a sliding window with a kmer size of 28, step size of 1. Screen reads were trimmed from both ends using a minimum quality cutoff of 3, reads with 3 or more s or with average quality score of less than Q20 were removed. In addition, reads with a minimum sequence length of <50 bps were removed.

Contig binning

Bowtie 2 [13] version 2.1.0 (bowtie-bio.sourceforge.net/bowtie2/index.shtml) and MarkDuplicates from Picard tools version 1.77 (picard.sourceforge.net/) were used (with default parameters) to map quality trimmed reads from all 16 samples onto the contigs of the co-assembly, in order to calculate contig coverages across samples. Only contigs > 5000 bp were used as input for CONCOCT [14] version 0.2 (github.com/BinPro/CONCOCT). After binning, some bins were subdivided based on their coverage pattern across samples (Supplementary Data 2). This concerns bin 4 divided in 4a, 4b and 4c, bin 7 divided in 7a and 7b, bin 8 divided in 8a, 8b and 8c, bin 16 divided in 16a and 16b, bin 20 divided in 20a and 20b and bin 40 divided in 40 and 40x (composed of only one contig). Purity and completeness of each bin was then assessed using CheckM [15] v. 0.9.4 (github.com/Ecogenomics/CheckM) using the lineage-specific workflow. Bins with completeness greater than 75% and a

contamination smaller than 10% were considered as draft genomes that are high- quality and nearly complete.

Taxonomic annotation of bins

For taxonomic affiliation of genomes, the following method was applied. If a 16S rRNA gene was detected using Barrnap version 0.4.2 (www.vicbioinformatics.com/software.barrnap.shtml), the annotation of RDP classifier [16] version 2.7 (sourceforge.net/projects/rdp-classifier/) was used with a confidence threshold of 0.8. If no 16S rRNA gene could be detected, MLtreeMap [17] version 2.061 (mltreemap.org/) annotations were used instead, choosing the taxonomic level that contains at least 66.67% of all marker genes (arbitrary cut-off). When the default and geba tree produced results with different taxonomic levels, the highest one was chosen. If all marker genes were affiliated to a single species, the highest taxonomic level that is not shared by adjacent species on the tree was chosen. IMG [18] phylogenetic distributions (img.jgi.doe.gov/mer/) were not used for assessing the taxonomic annotation of genomes.

The average nucleotide identity (ANI) between the selected draft genomes and references genomes found in NCBI genome database (www.ncbi.nlm.nih.gov/genome/) was calculated using the default parameters of the Kostas Lab's ANI calculator (enve-omics.ce.gatech.edu/ani/). For selecting reference genomes, a BLASTN [10] analysis of the 16S rRNA gene of the draft genomes was carried out on NCBI (blast.ncbi.nlm.nih.gov/blast.cgi), using as database the 16S ribosomal RNA sequences database. Depending on the outcome, either the 15 top BLASTN hits, or all genomes belonging to a genus or family, were used as references genomes.

Further manual binning corrections

Because bins 40x and 53 have complementary single-copy genes based on CheckM output (results not shown) and have similar annotation output results, it was decided to merge them into a newly defined bin 58. Bin 15 contained 10 different 16S rRNA genes. This can be explained by the difficulty of binning contigs harboring these conserved genes, because of specific tetranucleotides frequency patterns and because of biased coverage patterns. However, based on taxonomic annotation and coverage pattern comparison, these incorrectly annotated contigs could be transferred to their correct bin. This concerns contig BRHa_1003273 placed in bin 11, contig BRHa_1000755 placed in bin 35, contig BRHa_1003404 placed in bin 41, contig BRHa_1000455 placed in bin 46, contig BRHa_1007118 placed in bin 52, contig BRHa_1004490 placed in bin 20a, and contig

BRHa_1004053 placed in bin 8a (Supplementary Data 2). Contig BRHa_1000758 was classified as contaminating sequence since it perfectly matches PhIX phage genome. The first 40 nucleotides of BRHa_1006152 were trimmed off because they match an adaptor used for sequencing processes.

Pathway annotation of bins

Genes were annotated with the IMG pipeline [18] (img.jgi.doe.gov/). Metabolic pathways were manually annotated using in-house databases based on KEGG [19] (www.genome.jp/kegg/) and MetaCyc [20] (metacyc.org/) databases, and textbook biochemical pathways [21]. In order to determine the subcellular localization of proteins, PSORTb [22] version 3.0.2 (www.psort.org/psortb/) was used. For comparing some gene cluster at the synteny level, BLAST [10] version 2.2.28 (www.ncbi.nlm.nih.gov/books/NBK1762/) and Mauve [23] version 2.3.1 (asap.genetics.wisc.edu/software/mauve/) were used.

Proportion of microorganisms in microbial community

The abundance $a(B_i)$ of each bin B_i greater than 500 kb in size compared the abundance of the entire microbial community M is defined as follows:

$$M = \sum_{i=1}^{m} a(B_i)$$

where m is the number of bins and

$$a(B_i) = \frac{\sum_{j=1}^{n} l(C_j) \cdot c(C_j)}{\sum_{j=1}^{n} l(C_j)}$$

where n is the number of contigs in bin B_i, $c(C_j)$ the mean coverage of C_j and $l(C_j)$ is the length of contig C_j. The abundance of a bin B_i is then expressed as its mean coverage. The relative abundance $r(B_i)$, or contribution, of each bin was defined as

$$r(B_i) = \frac{a(B_i)}{M}$$

Metaproteomics

Two borehole water samples (0.45 L each) were collected and filtered by Sterivex 0.22 µm polyethersulfone membrane (Millipore, Billerica, USA) 483 days after the first H_2 injection and directly frozen in dry ice. The frozen filter was cut into small pieces, pooled together and immersed in detergent based lysis buffer, described by Chourey et al. [24]. The cells trapped on the filters were heat-lysed and processed as described earlier [25]. The cell lysate (supernatant) was aliquoted into fresh tubes and amended with chilled 100% trichloroacetic acid (TCA) to a

final concentration of 25% (vol/vol) and kept at -20°C overnight. The filter pieces were discarded. Following TCA precipitation, the cell lysate was centrifuged at 21,000 x *g* for 20 min to obtain a protein pellet. The pellet was retained and the supernatant discarded. The protein pellet was washed twice with chilled acetone as described earlier [30], dried at room temperature and solubilized in 6 M guanidine buffer (6 M guanidine; 10 mM dithiothreitol [DTT] in Tris-$CaCl_2$ buffer (50mM Tris; 10mM $CaCl_2$, pH 7.8) and incubated at 60°C for three hours with intermittent vortexing. An aliquot of 25 µl was utilized for protein concentration estimation, carried out using the RC/DC protein estimation kit (Bio-Rad Laboratories, Hercules, USA) as per the manufacturer's instructions. Total protein extracted from the borehole water was 250 µg. Following protein estimation results, rest of the protein sample was digested with modified sequencing grade trypsin overnight at 37°C, peptides desalted using seppak column and solvent exchanged as described earlier [26]. Peptides were stored at -80°C until MS analysis.

All chemicals used for sample processing, cleanup and mass spectrometry analysis were obtained from Sigma Chemical Co. (St. Louis, USA). Sequencing-grade trypsin was acquired from Promega (Madison, USA). Any other sources used to obtain the chemicals have been mentioned where required. High performance liquid chromatography- (HPLC-) grade water and other solvents were obtained from Burdick & Jackson (Muskegon, USA), 99% formic acid was purchased from EM Science (Darmstadt, Germany).

A single aliquot of 75 ug peptide mix was loaded onto a biphasic resin packed column [SCX (Luna, Phenomenex, Torrance, USA) and C18 (Aqua, Phenomenex, Torrance, USA)] as described earlier [26,27]. Following sample loading, the column was washed for 15 min, offline as described by Sharma (*28*) and connected to the C18 packed nanospray tip (New Objective, Woburn, USA) mounted on Proxeon (Odense, Denmark) nanospray source as described earlier [28]. Peptides were subjected to 24h multi-step chromatographic separation via the Ultimate 3000 HPLC system (Dionex, Sunnyvale, USA) connected to the mass spectrometer and measurements done using the Multi-Dimensional Protein Identification Technology (MuDPIT) approach as described earlier [26–28]. The peptide fragmentation was executed and recorded via an LTQ-Orbitrap-Elite mass spectrometer (ThermoFisher Scientific, Germany) operated in data dependent mode, via Thermo Xcalibur software V2.1.0. Each full scan (1 microscan) was followed by collision-activated dissociation (CID) based fragmentation using 35% collision energy of 20 most abundant parent ions (1 microscan) with a mass exclusion width of 0.2 m/z and dynamic exclusion duration of 60 s. The peptide sample was analyzed via MuDPIT as two independent runs (technical duplicates).

For protein identification, the raw spectra were searched against the protein database generated by groundwater sample sequencing (as described above), via Myrimatch v2.1 algorithm [29] (omictools.com/myrimatch-tool) set to parameters described previously [30] with minor modifications such as omission of static cysteine and dynamic oxidation modifications. Identification of at least two peptides per protein (one unique and one non-unique) sequence was set as a prerequisite for protein identification. Common contaminant peptide sequences from trypsin and keratin were concatenated to the database. Reverse database sequences were also included in the database as decoy sequences to calculate false discovery rate (FDR). False discovery rate (FDR) cutoff for peptide to spectrum identification was maintained at < 1%. For downstream data analysis, spectral counts of identified peptides was normalized as described before [31] to obtain the normalized spectral abundance factor (NSAF [31]), which was further adjusted by multiplying NSAF by 10^5 to obtain normalized spectral counts (nSpc). The sample was analyzed in duplicate, and the average nSpc values of technical duplicates was considered as the total proteome profile of borewater sample. The proteins were ranked in the order of high to low nSpc counts to indicate high to low protein abundances in the sample.

SUPPLEMENTARY REFERENCES

1. Wersin, P. *et al.* Biogeochemical processes in a clay formation in situ experiment: Part A – Overview, experimental design and water data of an experiment in the Opalinus Clay at the Mont Terri Underground Research Laboratory, Switzerland. *Appl. Geochem.* **26,** 931–953 (2011).
2. Cline, J. D. Spectrophotometric determination of hydrogen sulfide in natural waters. *Limnol. Oceanogr.* **14,** 454–458 (1969).
3. Stookey, L. L. Ferrozine - a new spectrophotometric reagent for iron. *Anal. Chem.* **42,** 779–781 (1970).
4. Sander, R. *Compilation of Henry's Law Constants for Inorganic and Organic Species of Potential Importance in Environmental Chemistry*. (Max-Planck Institute of Chemistry, Air Chemistry Department Mainz, Germany, 1999).
5. Lunau, M., Lemke, A., Walther, K., Martens-Habbena, W. & Simon, M. An improved method for counting bacteria from sediments and turbid environments by epifluorescence microscopy. *Environ. Microbiol.* **7,** 961–968 (2005).
6. Tremblay, J. *et al.* Primer and platform effects on 16S rRNA tag sequencing. *Evol. Genomic Microbiol.* 771 (2015). doi:10.3389/fmicb.2015.00771
7. Edgar, R. C. Search and clustering orders of magnitude faster than BLAST. *Bioinformatics* **26,** 2460–2461 (2010).
8. Edgar, R. C. UPARSE: highly accurate OTU sequences from microbial amplicon reads. *Nat. Methods* **10,** 996–998 (2013).
9. Edgar, R. C., Haas, B. J., Clemente, J. C., Quince, C. & Knight, R. UCHIME improves sensitivity and speed of chimera detection. *Bioinformatics* **27,** 2194–2200 (2011).
10. Altschul, S. F., Gish, W., Miller, W., Myers, E. W. & Lipman, D. J. Basic local alignment search tool. *J. Mol. Biol.* **215,** 403–410 (1990).
11. Joshi, N. A. & Fass, J. N. *Sickle: A sliding-window, adaptive, quality-based trimming tool for FastQ files*. (2011).
12. Boisvert, S., Raymond, F., Godzaridis, É., Laviolette, F. & Corbeil, J. Ray Meta: scalable de novo metagenome assembly and profiling. *Genome Biol.* **13,** R122 (2012).
13. Langmead, B. & Salzberg, S. L. Fast gapped-read alignment with Bowtie 2. *Nat. Methods* **9,** 357–359 (2012).
14. Alneberg, J. *et al.* Binning metagenomic contigs by coverage and composition. *Nat. Methods* **11,** 1144–1146 (2014).

15. Parks, D. H., Imelfort, M., Skennerton, C. T., Hugenholtz, P. & Tyson, G. W. CheckM: assessing the quality of microbial genomes recovered from isolates, single cells, and metagenomes. *Genome Res.* gr.186072.114 (2015). doi:10.1101/gr.186072.114
16. Wang, Q., Garrity, G. M., Tiedje, J. M. & Cole, J. R. Naive Bayesian classifier for rapid assignment of rRNA sequences into the new bacterial taxonomy. *Appl. Environ. Microbiol.* **73,** 5261–5267 (2007).
17. Stark, M., Berger, S. A., Stamatakis, A. & von Mering, C. MLTreeMap - accurate maximum likelihood placement of environmental DNA sequences into taxonomic and functional reference phylogenies. *BMC Genomics* **11,** 461 (2010).
18. Markowitz, V. M. *et al.* IMG 4 version of the integrated microbial genomes comparative analysis system. *Nucleic Acids Res.* **42,** D560–D567 (2014).
19. Kanehisa, M. *et al.* Data, information, knowledge and principle: back to metabolism in KEGG. *Nucleic Acids Res.* **42,** D199–D205 (2014).
20. Caspi, R. *et al.* The MetaCyc database of metabolic pathways and enzymes and the BioCyc collection of pathway/genome databases. *Nucleic Acids Res.* **42,** D459–D471 (2014).
21. Kim, B. H. & Gadd, G. M. *Bacterial Physiology and Metabolism*. (Cambridge University Press, 2008).
22. Yu, N. Y. *et al.* PSORTb 3.0: improved protein subcellular localization prediction with refined localization subcategories and predictive capabilities for all prokaryotes. *Bioinformatics* **26,** 1608–1615 (2010).
23. Darling, A. E., Mau, B. & Perna, N. T. Progressive Mauve: Multiple alignment of genomes with gene flux and rearrangement. *ArXiv09105780 Q-Bio* (2009).
24. Chourey, K. *et al.* Direct cellular lysis/protein extraction protocol for soil metaproteomics. *J. Proteome Res.* **9,** 6615–6622 (2010).
25. Chourey, K. *et al.* Environmental proteomics reveals early microbial community responses to biostimulation at a uranium- and nitrate-contaminated site. *Proteomics* **13,** 2921–2930 (2013).
26. Thompson, M. R. *et al.* Dosage-dependent proteome response of Shewanella oneidensis MR-1 to acute chromate challenge. *J. Proteome Res.* **6,** 1745–1757 (2007).
27. Brown, S. D. *et al.* Molecular dynamics of the Shewanella oneidensis response to chromate stress. *Mol. Cell. Proteomics MCP* **5,** 1054–1071 (2006).
28. Sharma, R. *et al.* Coupling a detergent lysis/cleanup methodology with intact protein fractionation for enhanced proteome characterization. *J. Proteome Res.* **11,** 6008–6018 (2012).

29. Tabb, D. L., Fernando, C. G. & Chambers, M. C. MyriMatch: highly accurate tandem mass spectral peptide identification by multivariate hypergeometric analysis. *J. Proteome Res.* **6,** 654–661 (2007).

30. Xiong, W., Giannone, R. J., Morowitz, M. J., Banfield, J. F. & Hettich, R. L. Development of an enhanced metaproteomic approach for deepening the microbiome characterization of the human infant gut. *J. Proteome Res.* **14,** 133–141 (2015).

31. Paoletti, A. C. *et al.* Quantitative proteomic analysis of distinct mammalian Mediator complexes using normalized spectral abundance factors. *Proc. Natl. Acad. Sci. U. S. A.* **103,** 18928–18933 (2006).
